# Studying Asymmetric Structure in Directed Networks by Overlapping and Non-Overlapping Models

**DOI:** 10.3390/e24091216

**Published:** 2022-08-30

**Authors:** Huan Qing

**Affiliations:** School of Mathematics, China University of Mining and Technology, Xuzhou 221116, China; qinghuan@cumt.edu.cn

**Keywords:** community detection, directed network, network analysis, spectral clustering

## Abstract

We consider the problem of modeling and estimating communities in directed networks. Models to this problem in the previous literature always assume that the sending clusters and the receiving clusters have non-overlapping property or overlapping property simultaneously. However, previous models cannot model the directed network in which nodes in sending clusters have overlapping property, while nodes in receiving clusters have non-overlapping property, especially for the case when the number of sending clusters is no larger than that of the receiving clusters. This kind of directed network exists in the real world for its randomness, and by the fact that we have little prior knowledge of the community structure for some real-world directed networks. To study the asymmetric structure for such directed networks, we propose a flexible and identifiable Overlapping and Non-overlapping model (ONM). We also provide one model as an extension of ONM to model the directed network, with a variation in node degree. Two spectral clustering algorithms are designed to fit the models. We establish a theoretical guarantee on the estimation consistency for the algorithms under the proposed models. A small scale computer-generated directed networks are designed and conducted to support our theoretical results. Four real-world directed networks are used to illustrate the algorithms, and the results reveal the existence of highly mixed nodes and the asymmetric structure for these networks.

## 1. Introduction

Community detection is a powerful tool in studying social networks with a latent structure of community [1,2,3,4]. The goal of community detection is to estimate a node’s community information from the network. In the study of social networks, various models have been proposed for community detection to model different networks with different community structures [5]. Due to the extremely intensive studies on community detection, we only focus on identifiable models that are closely relevant to our study in this paper.

The Stochastic Blockmodel (SBM) [6] is a classical and widely used model for an undirected network. SBM assumes that the probability of an edge between two nodes only depends on the clusters they belong to, and this assumption is not realistic because nodes have various degrees in real-world networks. To model real-world un-directed networks in which nodes degrees vary, the Degree-Corrected Stochastic Blockmodel (DCSBM) [7] extends SBM by introducing degree heterogeneities. Under SBM and DCSBM, all nodes are pure, such that each node only belongs to one community. However, in real cases, some nodes may belong to multiple communities, and such nodes have overlapping (also known as mixed membership) property. To model undirected networks in which nodes have an overlapping property, Ref. [8] designs the Mixed Membership Stochastic Blockmodel (MMSB). Ref. [9] introduces the Degree-Corrected Mixed Membership model (DCMM), which extends MMSB by considering degree heterogeneities. Ref. [10] designs the Overlapping Continuous Community Assignment model (OCCAM), which equals DCMM actually. Spectral methods with consistent estimations under the above models are provided in [9,11,12,13,14,15,16,17].

For directed networks in which all nodes have a non-overlapping property, Ref. [18] proposes a model called Stochastic co-Blockmodel, (ScBM) and its extension, the Degree-Corrected Stochastic co-Blockmodel (DCScBM), by considering the degree heterogeneity, where ScBM (DCScBM) is an extension of SBM (DCSBM) from an un-directed network to a directed network. ScBM and DCScBM can model non-overlapping directed networks in which row nodes belong to $K_{r}$ sending clusters (we also use community to denote cluster occasionally) and column nodes belong to $K_{c}$ receiving clusters, where row nodes can differ from column nodes, and $K_{r}$ can differ from $K_{c}$. Ref. [19] studies the consistency of some adjacency-based spectral algorithms under ScBM. Ref. [20] studies the consistency of the spectral method D-SCORE under DCScBM when $K_{r}=K_{c}$. Ref. [21] designs the Directed Mixed Membership Stochastic Blockmodel (DiMMSB) as an extension of ScBM and MMSB to model directed networks in which all nodes have overlapping property. Meanwhile, DiMMSB can also be seen as an extension of the two-way blockmodels with a Bernoulli distribution of [22]. All of the above models are identifiable under certain conditions. The identifiability of ScBM and DCScBM holds even for the case when $K_{r}\neq K_{c}$. DiMMSB is identifiable only when $K_{r}=K_{c}$. Sure, SBM, DCSBM, MMSB, DCMM, and OCCAM are identifiable when $K_{r}=K_{c}$, since they model undirected networks. For all the above models, row nodes and column nodes have symmetric structural information such that they always have non-overlapping property or overlapping property simultaneously. As shown by the identifiability of DiMMSB, to model a directed network in which all nodes have overlapping property, the identifiability of the model requires $K_{r}=K_{c}$. Naturally, there is a bridge model from ScBM to DiMMSB such that the bride model can model a directed network in which the row nodes and column nodes have asymmetric structural information such that they have different overlapping property. In this paper, we introduce this model and name it the Overlapping and Non-overlapping model.

Our contributions in this paper are as follows. We propose an identifiable model for directed networks, the Overlapping and Non-overlapping model (ONM for short). ONM allows that nodes in a directed network can have different overlapping properties. Without a loss of generality, in a directed network, we let the row nodes have overlapping property while the column nodes do not. The proposed model is identifiable when $K_{r}\leq K_{c}$. Recall that the identifiability of ScBM modeling non-overlapping directed networks holds even for the case $K_{r}\neq K_{c}$, and that DiMMSB modeling overlapping directed networks is identifiable only when $K_{r}=K_{c}$, this is the reason for why we call ONM modeling directed networks, in which row nodes have different overlapping properties to column nodes, as a bridge model from ScBM to DiMMSB. We also propose an identifiable model, Overlapping and Degree-Corrected Non-overlapping model (ODCNM), as an extension of ONM, by considering the degree heterogeneity. We construct two spectral algorithms to fit ONM and ODCNM. We show that our methods enjoy consistent estimations under mild conditions. Especially, our theoretical results under ODCNM match those under ONM when ODCNM reduces to ONM. The numerical results of simulated directed networks generated under ONM and ODCNM support our theoretical findings, and the results on four real-world directed networks demonstrate the advantages of our algorithms in studying the asymmetric structure between the sending and receiving clusters.

*Notations.* We take the following general notations in this paper. For any positive integer *m*, let [m]:={1,2,…,m}, and let $I_{m}$ denote the m×m identity matrix. For a vector *x* and fixed q>0, ${∥x∥}_{q}$ denotes its $l_{q}$-norm. For a matrix *M*, $M^{\prime }$ denotes the transpose of the matrix *M*, ∥M∥ denotes the spectral norm, ${∥M∥}_{F}$ denotes the Frobenius norm, and ${∥M∥}_{2\to \infty }$ denotes the maximum $l_{2}$-norm of all the rows of *M*. Let $\sigma_{i}\left(M\right)$ be the *i*-th largest singular value of matrix *M*, and let $\lambda_{i}\left(M\right)$ denote the *i*-th largest eigenvalue of the matrix *M* ordered by the magnitude. M(i,:) and M(:,j) denote the *i*-th row and the *j*-th column of matrix *M*, respectively. $M(S_{r},:)$ and $M(:,S_{c})$ denote the rows and columns in the index sets $S_{r}$ and $S_{c}$ of matrix *M*, respectively. For any matrix *M*, we simply use Y=max(0,M) to represent $Y_{ij}=\max\left(0,M_{ij}\right)$ for any i,j. For any matrix $M\in R^{m\times m}$, let diag(M) be the m×m diagonal matrix whose *i*-th diagonal entry is M(i,i), and let rank(M) be *M*’s rank. 1 is a column vector with all entries being the value 1. $e_{i}$ is a column vector whose *i*-th entry is 1, while other entries are zero. In this paper, *C* is a positive constant which may occasionally be different.

## 2. The Overlapping and Non-Overlapping Model

Consider a directed network $N=(V_{r},V_{c},E)$, where $V_{r}=\left\{1,2,\ldots ,n_{r}\right\}$ is the set of row nodes, $V_{c}=\left\{1,2,\ldots ,n_{c}\right\}$ is the set of column nodes, and *E* is the set of edges from the row nodes to the column nodes. Note that since the row nodes can be different from the column nodes, we may have $V_{r}\cap V_{c}=⌀$ (i.e., there are no common nodes between $V_{r}$ and $V_{c}$), and $V_{r}$ may not be equal to $V_{c}$ (i.e., the row nodes are different from the column nodes), which is a more general case than $V_{r}=V_{c}$ (i.e., all row nodes are same as column nodes), where ⌀ denotes the null set, and such a directed network N is also known as a bipartite graph (or bipartite network) in [18,19]. In this paper, we use the subscript *r* and *c* to distinguish the terms for the row nodes and column nodes, where works in [18,19,23,24,25,26] also consider the general bipartite setting, such that the row nodes may differ from the column nodes. Let $A\in {\left\{0,1\right\}}^{n_{r}\times n_{c}}$ be the bi-adjacency matrix of directed network N, such that $A(i_{r},i_{c})=1$ if there is a directional edge from row node $i_{r}$ to column node $i_{c}$, and $A(i_{r},i_{c})=0$ otherwise. For convenience, we call the community that the row nodes belong to as the row community (or sending cluster occasionally), and the community that the column nodes belong to as the column community (or receiving cluster occasionally).

We propose a new blockmodel which we call the Overlapping and Non-overlapping model (ONM for short). ONM can model directed networks whose row nodes belong to $K_{r}$ overlapping row communities, while the column nodes belong to $K_{c}$ non-overlapping column communities. For row nodes, let $\Pi_{r}\in R^{n_{r}\times K_{r}}$ be the membership matrix, such that

(1) $$\begin{array}{c} \Pi_{r}\left(i_{r},\right)\geq 0,{∥\Pi_{r}\left(i_{r},:\right)∥}_{1}=1\;\mathrm{for}\;i_{r}\in \left[n_{r}\right]. \end{array}$$

Call row node $i_{r}$ *pure* if $\Pi_{r}\left(i_{r},:\right)$ degenerates (i.e., one entry is 1, all others $K_{r}-1$ entries are 0), and *mixed* otherwise. From such a definition, row node $i_{r}$ has mixed membership and may belong to more than one row communities for $i_{r}\in \left[n_{r}\right]$.

For column nodes, let *ℓ* be the $n_{c}\times 1$ vector whose $i_{c}$-th entry $\ell (i_{c})=k$ if column node $i_{c}$ belongs to the *k*-th column community, and $\ell (i_{c})$ takes value from $\left\{1,2,\ldots ,K_{c}\right\}$ for $i_{c}\in \left[n_{c}\right]$. Let $\Pi_{c}\in R^{n_{c}\times K_{c}}$ be the membership matrix of column nodes, such that for $i_{c}\in \left[n_{c}\right],k\in \left[K_{c}\right]$,

(2) $$\begin{array}{c} \Pi_{c}\left(i_{c},k\right)=1\;\mathrm{when}\;\ell \left(i_{c}\right)=k,\;\mathrm{and}\;0\;\mathrm{otherwise},\mathrm{and}\;{∥\Pi_{c}\left(i_{c},:\right)∥}_{1}=1. \end{array}$$

From such a definition, column node $i_{c}$ belongs to *exactly* one of the $K_{c}$ column communities for $i_{c}\in \left[n_{c}\right]$. Sure, all of the column nodes are pure nodes.

In this paper, we assume that

(3) $$\begin{array}{c} K_{r}\leq K_{c}. \end{array}$$

Equation (3) is required for the identifiability of ONM. Let $P\in R^{K_{r}\times K_{c}}$ be the probability matrix, such that

(4) $$\begin{array}{c} 0\leq P\left(k,l\right)\leq \rho \leq 1\;\mathrm{for}\;k\in \left[K_{r}\right],l\in \left[K_{c}\right], \end{array}$$

where ρ controls the network sparsity and is called the sparsity parameter in this paper. For convenience, set $P=\rho \tilde{P}$, where $\tilde{P}\left(k,l\right)\in \left[0,1\right]$ for $k\in \left[K_{r}\right],l\in \left[K_{c}\right]$, and $\max_{k\in \left[K_{r}\right],l\in \left[K_{c}\right]}\tilde{P}\left(k,l\right)=1$ for model identifiability. For all pairs of $\left(i_{r},i_{c}\right)$ with $i_{r}\in \left[n_{r}\right],i_{c}\in \left[n_{c}\right]$, our model assumes that $A(i_{r},i_{c})$ are independent Bernoulli random variables satisfying

(5) $$\begin{array}{c} \Omega :=\Pi_{r}P\Pi_{c}^{\prime },\;\;\;A\left(i_{r},i_{c}\right)∼\mathrm{Bernoulli}\left(\Omega \left(i_{r},i_{c}\right)\right), \end{array}$$

where Ω=E[A], and we call it the population adjacency matrix in this paper.

**Definition** **1.** *Call model (1)–(5) the Overlapping and Non-overlapping model (ONM), and denote it with $ONM_{n_{r},n_{c}}\left(K_{r},K_{c},P,\Pi_{r},\Pi_{c}\right)$.*

**Remark** **1.** *Under $ONM_{n_{r},n_{c}}\left(K_{r},K_{c},P,\Pi_{r},\Pi_{c}\right)$, for $i_{r}\in \left[n_{r}\right],j_{c}\in \left[n_{c}\right]$, since $P\left(A\left(i_{r},j_{c}\right)=1\right)=\Omega \left(i_{r},j_{c}\right)=\rho \Pi \left(i_{r},:\right)\tilde{P}\Pi_{c}^{\prime }\left(j_{c},:\right)$, we see that increasing ρ increases the probability to generate an edge from row node $i_{r}$ to column node $j_{c}$, i.e., the sparsity of the network is governed by ρ.*

The following conditions are sufficient for the identifiability of ONM:

- (I1) $\mathrm{rank}\left(P\right)=K_{r},\mathrm{rank}\left(\Pi_{r}\right)=K_{r}$, and $\mathrm{rank}\left(\Pi_{c}\right)=K_{c}$.
- (I2) There is at least one pure row node for each of the $K_{r}$ row communities.

For $k\in [K_{r}]$, let $I_{r}^{\left(k\right)}=\left\{i\in \left[n_{r}\right]\right\}:\Pi_{r}\left(i,k\right)=1\}$. By condition (I2), $I_{r}^{\left(k\right)}$ is non-empty for all $k\in [K_{r}]$. For $k\in [K_{r}]$, select one row node from $I_{r}^{\left(k\right)}$ to construct the index set $I_{r}$; i.e., $I_{r}$ is the indices of row nodes corresponding to $K_{r}$ pure row nodes, one from each row community. Without loss of generality, let $\Pi_{r}\left(I_{r},:\right)=I_{K_{r}}$ (Lemma 2.1 [17] also has a similar setting to design their spectral algorithm under MMSB). $I_{c}$ is defined similarly for the column nodes, such that $\Pi_{c}\left(I_{c},:\right)=I_{K_{c}}$. The next proposition guarantees the identifiability of ONM.

**Proposition** **1.** *If conditions (I1) and (I2) hold, ONM is identifiable: For eligible $\left(P,\Pi_{r},\Pi_{c}\right)$ and $\left(\overset{ˇ}{P},{\overset{ˇ}{\Pi }}_{r},{\overset{ˇ}{\Pi }}_{c}\right)$, if $\Pi_{r}P\Pi_{c}^{\prime }={\overset{ˇ}{\Pi }}_{r}\overset{ˇ}{P}{\overset{ˇ}{\Pi }}_{c}^{\prime }$, then $P=\overset{ˇ}{P},\Pi_{r}={\overset{ˇ}{\Pi }}_{r}$, and $\Pi_{c}={\overset{ˇ}{\Pi }}_{c}$.*

All proofs of propositions, lemmas, and theorems are provided in Appendix B and Appendix C of this paper. Compared to some previous models, ONM models different directed networks.

- When the row nodes are the same as the column nodes, $K_{r}=K_{c}$, and all nodes are pure, ONM degenerates to SBM. However, ONM can model directed networks where row nodes enjoy mixed memberships, while SBM only models un-directed networks.
- When all row nodes are pure, our ONM reduces to ScBM with $K_{r}$ row clusters and $K_{c}$ column clusters [18]. However, ONM allows for row nodes to have overlapping memberships, while ScBM does not. Meanwhile, for model identifiability, ScBM does not require $\mathrm{rank}\left(P\right)=K_{r}$ that ONM requires, and this can be seen as the cost of ONM when modeling the overlapping row nodes.
- Though DiMMSB [21] can model directed networks whose row and column nodes have overlapping memberships, DiMMSB requires $K_{r}=K_{c}$ for model identifiability. For comparison, our ONM allows $K_{r}\leq K_{c}$ at the cost of losing the overlapping property of the column nodes.

### 2.1. A Spectral Algorithm for Fitting ONM

The primary goal of the proposed algorithm is to estimate the row membership matrix $\Pi_{r}$ and the column membership matrix $\Pi_{c}$ from the observed adjacency matrix *A* with a given $K_{r}$ and $K_{c}$. We now discuss our intuition for the design of our algorithm to fit ONM.

Under conditions (I1) and (I2), by basic algebra, we have $\mathrm{rank}\left(\Omega \right)=K_{r}$. Let $\Omega =U_{r}\Lambda U_{c}^{\prime }$ be the compact singular value decomposition of Ω, where $U_{r}\in R^{n_{r}\times K_{r}},\Lambda \in R^{K_{r}\times K_{r}},U_{c}\in R^{n_{c}\times K_{r}}$, $U_{r}^{\prime }U_{r}=I_{K_{r}},U_{c}^{\prime }U_{c}=I_{K_{r}}$, and $I_{K_{r}}$ is a $K_{r}\times K_{r}$ identity matrix. Let $n_{c,k}=\left|\left\{i_{c}:\ell \left(i_{c}\right)=k\right\}\right|$ be the size of the *k*-th column community for $k\in [K_{c}]$. Let $n_{c,\max}=\max_{k\in [K_{c}]}n_{c,k}$ and $n_{c,\min}=\min_{k\in [K_{c}]}n_{c,k}$. Meanwhile, without causing confusion, let $n_{c,K_{r}}$ be the $K_{r}$-th largest size among all column communities. The following lemma guarantees that $U_{r}$ enjoys an ideal simplex structure and $U_{c}$ has $K_{c}$ distinct rows.

**Lemma** **1.** *Under*$ONM_{n_{r},n_{c}}\left(K_{r},K_{c},P,\Pi_{r},\Pi_{c}\right)$, *there exists a unique*$K_{r}\times K_{r}$*matrix*$B_{r}$*and a unique*$K_{c}\times K_{r}$*matrix*$B_{c}$, *such that*

- *$U_{r}=\Pi_{r}B_{r}$, where $B_{r}=U_{r}\left(I_{r},:\right)$. Meanwhile, $U_{r}\left(i_{r},:\right)=U_{r}\left({\bar{i}}_{r},:\right)$ when $\Pi_{r}\left(i_{r},:\right)=\Pi_{r}\left({\bar{i}}_{r},:\right)$ for $i_{r},{\bar{i}}_{r}\in \left[n_{r}\right]$.*
- $U_{c}=\Pi_{c}B_{c}$. *Meanwhile,*$U_{c}\left(i_{c},:\right)=U_{c}\left({\bar{i}}_{c},:\right)$*when*$\ell \left(i_{c}\right)=\ell \left({\bar{i}}_{c}\right)$*for*$i_{c},{\bar{i}}_{c}\in \left[n_{c}\right]$, *i.e.,*$U_{c}$*has*$K_{c}$*distinct rows. Furthermore, when*$K_{r}=K_{c}=K$, *we have*$∥B_{c}\left(k,:\right)-B_{c}{\left(l,:\right)∥}_{F}=\sqrt{\frac{1}{n_{c,k}}+\frac{1}{n_{c,l}}}$*for all*1≤k<l≤K.

Lemma 1 says that the rows of $U_{c}$ form a $K_{r}$-simplex in $R^{K_{r}}$, which we call the Ideal Simplex (IS), with the $K_{r}$ rows of $B_{r}$ being the vertices. This IS is also found in [9,17,21]. Meanwhile, Lemma 1 says that $U_{c}$ has $K_{c}$ distinct rows, and if two column nodes $i_{c}$ and ${\bar{i}}_{c}$ are from the same column community, then $U_{c}\left(i_{c},:\right)=U_{c}\left({\bar{i}}_{c},:\right)$.

Under ONM, to recover $\Pi_{c}$ from $U_{c}$, since $U_{c}$ has $K_{c}$ distinct rows, applying the k-means algorithm on all rows of $U_{c}$ returns true column communities by Lemma 1. Since $U_{c}$ has $K_{c}$ distinct rows, we can set $\delta_{c}=\min_{k\neq l}{∥B_{c}\left(k,:\right)-B_{c}\left(l,:\right)∥}_{F}$ to measure the minimum center separation of $B_{c}$. By Lemma 1, $\delta_{c}\geq \sqrt{\frac{2}{n_{c,\max}}}$ when $K_{r}=K_{c}=K$ under $ONM_{n_{r},n_{c}}\left(K_{r},K_{c},P,\Pi_{r},\Pi_{c}\right)$. However, when $K_{r}<K_{c}$, it is a challenge to obtain a positive lower bound of $\delta_{c}$; see the proof of Lemma 1 for details.

Under ONM, to recover $\Pi_{r}$ from $U_{r}$, since $B_{r}$ is full rank, if $U_{r}$ and $B_{r}$ are known in advance ideally, we can exactly recover $\Pi_{r}$ by setting $\Pi_{r}=U_{r}B_{r}^{\prime }{\left(B_{r}B_{r}^{\prime }\right)}^{-1}$ via Lemma 1. Set $Y_{r}=U_{r}B_{r}^{\prime }{\left(B_{r}B_{r}^{\prime }\right)}^{-1}$. Since $Y_{r}\equiv \Pi_{r}$ and $∥\Pi_{r}\left(i_{r},:\right){∥}_{1}=1$ for $i_{r}\in \left[n_{r}\right]$, we have

$$\begin{array}{c} \Pi_{r}\left(i_{r},:\right)=\frac{Y_{r}\left(i_{r},:\right)}{∥Y_{r}\left(i_{r},:\right){∥}_{1}},i_{r}\in \left[n_{r}\right]. \end{array}$$

With a given $U_{r}$, since it enjoys IS structure $U_{r}=\Pi_{r}B_{r}\equiv \Pi_{r}U_{r}\left(I_{r},:\right)$, as long as we can obtain the row corner matrix $U_{r}\left(I_{r},:\right)$ (i.e., $B_{r}$), we can recover $\Pi_{r}$ exactly. As mentioned in [9,17,21], for such an ideal simplex, the successive projection (SP) algorithm [27] (for details of SP, see Algorithm A1) can be applied to $U_{r}$ with $K_{r}$ row communities to find $U_{r}\left(I_{r},:\right)$.

Based on the above analysis, we are now ready to give the following algorithm which we call Ideal ONA. Input $\Omega ,K_{r}$, and $K_{c}$ with $K_{r}\leq K_{c}$. Outputs: $\Pi_{r}$ and *ℓ*.

- Let $\Omega =U_{r}\Lambda U_{c}^{\prime }$ be the compact SVD of Ω, such that $U_{r}\in R^{n_{r}\times K_{r}},U_{c}\in R^{n_{c}\times K_{r}},\Lambda \in R^{K_{r}\times K_{r}},U_{r}^{\prime }U_{r}=I_{K_{r}},and\;U_{c}^{\prime }U_{c}=I_{K_{r}}$.
- For the row nodes,
  - Run the SP algorithm on all rows of $U_{r}$, assuming there are $K_{r}$ row communities to obtain $U_{r}\left(I_{r},:\right)$. Set $B_{r}=U_{r}\left(I_{r},:\right)$.
  - Set $Y_{r}=U_{r}B_{r}^{\prime }{\left(B_{r}B_{r}^{\prime }\right)}^{-1}$. Recover $\Pi_{r}$ by setting $\Pi_{r}\left(i_{r},:\right)=\frac{Y_{r}\left(i_{r},:\right)}{∥Y_{r}\left(i_{r},:\right){∥}_{1}}$ for $i_{r}\in \left[n_{r}\right]$.
  For the column nodes,
  - Run k-means on $U_{c}$ assuming that there are $K_{c}$ column communities, i.e., find the solution to the following optimization problem
    $$\begin{array}{c} M^{*}={\mathrm{argmin}}_{M\in M_{n_{c},K_{r},K_{c}}}{∥M-U_{c}∥}_{F}^{2}, \end{array}$$
    where $M_{n_{c},K_{r},K_{c}}$ denotes the set of $n_{c}\times K_{r}$ matrices with only $K_{c}$ different rows.
  - Use $M^{*}$ to obtain the labels vector *ℓ* of the column nodes. Note that since $M^{*}$ has $K_{c}$ distinct rows, two different column nodes, $i_{c},{\bar{i}}_{c}\in \left[n_{c}\right]$, are in the same column community if $M^{*}\left(i_{c},:\right)=M^{*}\left({\bar{i}}_{c},:\right)$.

Following a similar proof of Theorem 1 of [21], the Ideal ONA exactly recovers row nodes memberships and column nodes labels, and this also verifies the identifiability of ONM in turn. For convenience, call the two steps for column nodes “run k-means on $U_{c}$ assuming there are $K_{c}$ column communities to obtain *ℓ*”.

We now extend the ideal case to the real case. Set $\tilde{A}={\hat{U}}_{r}\hat{\Lambda }{\hat{U}}_{c}^{\prime }$ be the top-$K_{r}$-dimensional SVD of *A*, such that ${\hat{U}}_{r}\in R^{n_{r}\times K_{r}},{\hat{U}}_{c}\in R^{n_{c}\times K_{r}},\hat{\Lambda }\in R^{K_{r}\times K_{r}},{\hat{U}}_{r}^{\prime }{\hat{U}}_{r}=I_{K_{r}},{\hat{U}}_{c}^{\prime }{\hat{U}}_{c}=I_{K_{r}}$, and $\hat{\Lambda }$ contains the top $K_{r}$ singular values of *A*. For the real case, we use ${\hat{B}}_{r},{\hat{B}}_{c},{\hat{Y}}_{r},{\hat{\Pi }}_{r},{\hat{\Pi }}_{c}$ given in Algorithm 1 to estimate $B_{r},B_{c},Y_{r},\Pi_{r},\Pi_{c}$, respectively. Algorithm 1, called the Overlapping and Non-overlapping algorithm (ONA for short), is a natural extension of the Ideal ONA to the real case. In ONA, we set the negative entries of ${\hat{Y}}_{r}$ as 0 by setting ${\hat{Y}}_{r}=\max\left(0,{\hat{Y}}_{r}\right)$, for the reason that the weights for any row node should be non-negative while there may exist some negative entries of ${\hat{U}}_{r}{\hat{B}}_{r}^{\prime }{\left({\hat{B}}_{r}{\hat{B}}_{r}^{\prime }\right)}^{-1}$. Note that in a directed network, if the column nodes have an overlapping property while row nodes do not, to perform community detection for such a directed network, the transpose of the adjacency matrix should be set as input when applying our algorithm.
**Algorithm 1Overlapping and Non-overlapping Algorithm** (**ONA**)**Require:** The adjacency matrix $A\in R^{n_{r}\times n_{c}}$ of a directed network, the number of row communities $K_{r}$, and the number of column communities $K_{c}$ with $K_{r}\leq K_{c}$.**Ensure:** The estimated $n_{r}\times K_{r}$ membership matrix ${\hat{\Pi }}_{r}$ for row nodes, and the estimated $n_{c}\times 1$ labels vector $\hat{\ell }$ for column nodes.
1:Compute ${\hat{U}}_{r}\in R^{n_{r}\times K_{r}}$ and ${\hat{U}}_{c}\in R^{n_{c}\times K_{r}}$ from the top-$K_{r}$-dimensional SVD of *A*.2:For row nodes:
Apply SP algorithm (i.e., Algorithm 2) on the rows of ${\hat{U}}_{r}$ assuming there are $K_{r}$ row clusters to obtain the near-corners matrix ${\hat{U}}_{r}\left({\hat{I}}_{r},:\right)\in R^{K_{r}\times K_{r}}$, where ${\hat{I}}_{r}$ is the index set returned by SP algorithm. Set ${\hat{B}}_{r}={\hat{U}}_{r}\left({\hat{I}}_{r},:\right)$.Compute the $n_{r}\times K_{r}$ matrix ${\hat{Y}}_{r}$ such that ${\hat{Y}}_{r}={\hat{U}}_{r}{\hat{B}}_{r}^{\prime }{\left({\hat{B}}_{r}{\hat{B}}_{r}^{\prime }\right)}^{-1}$. Set ${\hat{Y}}_{r}=\max\left(0,{\hat{Y}}_{r}\right)$ and estimate $\Pi_{r}\left(i_{r},:\right)$ by ${\hat{\Pi }}_{r}\left(i_{r},:\right)=\frac{{\hat{Y}}_{r}\left(i_{r},:\right)}{∥{\hat{Y}}_{r}\left(i_{r},:\right){∥}_{1}},i_{r}\in \left[n_{r}\right]$.  For column nodes: run k-means on ${\hat{U}}_{c}$ assuming there are $K_{c}$ column communities to obtain $\hat{\ell }$.

### 2.2. Main Results for ONA

In this section, we show the consistency of our algorithm for fitting the ONM as the number of row nodes $n_{r}$ and the number of column nodes $n_{c}$ increases. Throughout this paper, $K_{r}$ and $K_{c}$ are two known integers. First, we assume that:

**Assumption** **1.** *$\rho \max\left(n_{r},n_{c}\right)\geq \log\left(n_{r}+n_{c}\right)$.*

Assumption 1 controls the sparsity of the directed network considered for theoretical study. When building an estimation consistency of the spectral clustering methods in community detection, the sparsity assumption is common; see [13,14,17,18,20,21]. Especially, when ONM reduces to SBM, the sparsity requirement in Assumption 1 is consistent with that of Theorem 3.1 in [13], which guarantees the theoretical optimality on the sparsity condition of this paper. To measure the performance of ONA for row nodes memberships, since row nodes have mixed memberships, naturally, we use the $l_{1}$ norm difference between $\Pi_{r}$ and ${\hat{\Pi }}_{r}$. Since the column nodes are all pure nodes, we consider the performance criterion defined in [15] to measure the estimation error of ONA on the column nodes. We introduce this measurement of estimation error below.

Let $T_{c}=\left\{T_{c,1},T_{c,2},\ldots ,T_{c,K_{c}}\right\}$ be the true partition of column nodes $\left\{1,2,\ldots ,n_{c}\right\}$ obtained from *ℓ*, such that $T_{c,k}=\left\{i_{c}\in \left[n_{c}\right]:\ell \left(i_{c}\right)=k\right\}$ for $k\in [K_{c}]$. Let ${\hat{T}}_{c}=\left\{{\hat{T}}_{c,1},{\hat{T}}_{c,2},\ldots ,{\hat{T}}_{c,K_{c}}\right\}$ be the estimated partition of column nodes $\left\{1,2,\ldots ,n_{c}\right\}$ obtained from $\hat{\ell }$ of ONA, such that ${\hat{T}}_{c,k}=\left\{i_{c}\in \left[n_{c}\right]:\hat{\ell }\left(i_{c}\right)=k\right\}$ for $k\in [K_{c}]$. The criterion is defined as

$$\begin{array}{c} {\hat{f}}_{c}=\min_{\pi \in S_{K_{c}}}\max_{k\in [K_{c}]}\frac{|T_{c,k}\cap {\hat{T}}_{c,\pi (k)}^{c}\left|+\right|T_{c,k}^{c}\cap {\hat{T}}_{c,\pi (k)}|}{n_{c,k}}, \end{array}$$

where $S_{K_{c}}$ is the set of all permutations of $\left\{1,2,\ldots ,K_{c}\right\}$, and the superscript *c* denotes the complementary set. As mentioned in [15], ${\hat{f}}_{c}$ measures the maximum proportion of column nodes in the symmetric difference of $T_{c,k}$ and ${\hat{T}}_{c,\pi (k)}$.

The next theorem gives the theoretical bounds on the estimations of memberships for both the row and column nodes, which is the main theoretical result for ONA.

**Theorem** **1.** *Under $ONM_{n_{r},n_{c}}\left(K_{r},K_{c},P,\Pi_{r},\Pi_{c}\right)$, when Assumption 1 holds, suppose that $\sigma_{K_{r}}\left(\Omega \right)\geq C\sqrt{\rho \left(n_{r}+n_{c}\right)\log\left(n_{r}+n_{c}\right)}$, with a probability of at least $1-o({\left(n_{r}+n_{c}\right)}^{-\alpha })$ for any α>0,*

- *For row nodes, there exists a permutation matrix $P_{r}$ such that*
$$\begin{array}{cc} \; & \max_{i_{r}\in \left[n_{r}\right]}{∥e_{i_{r}}^{\prime }\left({\hat{\Pi }}_{r}-\Pi_{r}P_{r}\right)∥}_{1}=O\left(\varpi \kappa \left(\Pi_{r}^{\prime }\Pi_{r}\right)K_{r}\sqrt{\lambda_{1}\left(\Pi_{r}^{\prime }\Pi_{r}\right)}\right), \end{array}$$
  *where $\varpi =∥{\hat{U}}_{r}{\hat{U}}_{r}^{\prime }-U_{r}U_{r}^{\prime }{∥}_{2\to \infty }$ is the row-wise singular eigenvector error.*
- *For column nodes, ${\hat{f}}_{c}=O\left(\frac{K_{r}K_{c}\max\left(n_{r},n_{c}\right)\log\left(n_{r}+n_{c}\right)}{\sigma_{K_{r}}^{2}\left(\tilde{P}\right)\rho \delta_{c}^{2}\sigma_{K_{r}}^{2}\left(\Pi_{r}\right)n_{c,K_{r}}n_{c,\min}}\right)$. Especially, when $K_{r}=K_{c}=K$,*
$$\begin{array}{c} {\hat{f}}_{c}=O\left(\frac{K^{2}\max\left(n_{r},n_{c}\right)n_{c,\max}\log\left(n_{r}+n_{c}\right)}{\sigma_{K}^{2}\left(\tilde{P}\right)\rho \sigma_{K}^{2}\left(\Pi_{r}\right)n_{c,\min}^{2}}\right). \end{array}$$

Adding conditions similar to Corollary 3.1 in [17], we have the following corollary.

**Corollary** **1.** *Under $ONM_{n_{r},n_{c}}\left(K_{r},K_{c},P,\Pi_{r},\Pi_{c}\right)$, suppose conditions in Theorem 1 hold, and further, suppose that $\lambda_{K_{r}}\left(\Pi_{r}^{\prime }\Pi_{r}\right)=O\left(\frac{n_{r}}{K_{r}}\right),n_{c,\min}=O\left(\frac{n_{c}}{K_{c}}\right)$, with a probability of at least $1-o({\left(n_{r}+n_{c}\right)}^{-\alpha })$,*

- *For row nodes, when $K_{r}=K_{c}=K$,*
  $$\begin{array}{cc} \; & \max_{i_{r}\in \left[n_{r}\right]}{∥e_{i_{r}}^{\prime }\left({\hat{\Pi }}_{r}-\Pi_{r}P_{r}\right)∥}_{1}=O\left(\frac{K^{2}\left(\sqrt{\frac{C\max(n_{r},n_{c})}{\min(n_{r},n_{c})}}+\sqrt{\log(n_{r}+n_{c})}\right)}{\sigma_{K}\left(\tilde{P}\right)\sqrt{\rho n_{c}}}\right). \end{array}$$
- *For column nodes, ${\hat{f}}_{c}=O\left(\frac{K_{r}^{2}K_{c}^{3}\max\left(n_{r},n_{c}\right)\log\left(n_{r}+n_{c}\right)}{\sigma_{K_{r}}^{2}\left(\tilde{P}\right)\rho \delta_{c}^{2}n_{r}n_{c}^{2}}\right)$. When $K_{r}=K_{c}=K$,*
  $$\begin{array}{c} {\hat{f}}_{c}=O\left(\frac{K^{4}\max\left(n_{r},n_{c}\right)\log\left(n_{r}+n_{c}\right)}{\sigma_{K}^{2}\left(\tilde{P}\right)\rho n_{r}n_{c}}\right). \end{array}$$

*Especially, when*$n_{r}=O\left(n\right),n_{c}=O\left(n\right),K_{r}=O\left(1\right)$, *and*$K_{c}=O\left(1\right)$,

- *For row nodes, when $K_{r}=K_{c}=K$,*
  $$\begin{array}{c} \max_{i_{r}\in \left[n_{r}\right]}{∥e_{i_{r}}^{\prime }\left({\hat{\Pi }}_{r}-\Pi_{r}P_{r}\right)∥}_{1}=O\left(\frac{\sqrt{\log(n)}}{\sigma_{K}\left(\tilde{P}\right)\sqrt{\rho n}}\right). \end{array}$$
- *For column nodes, ${\hat{f}}_{c}=O\left(\frac{\log(n)}{\sigma_{K_{r}}^{2}\left(\tilde{P}\right)\rho \delta_{c}^{2}n^{2}}\right)$. When $K_{r}=K_{c}=K$,*
  $$\begin{array}{c} {\hat{f}}_{c}=O\left(\frac{\log(n)}{\sigma_{K}^{2}\left(\tilde{P}\right)\rho n}\right). \end{array}$$

When $n_{r}=O\left(n\right),n_{c}=O\left(n\right),K_{r}=K_{c}=K=O\left(1\right)$ in Corollary 1, the bounds for the row and column nodes are $O(\frac{1}{\sigma_{K}\left(\tilde{P}\right)}\sqrt{\frac{\log(n)}{n}})$ and $O(\frac{1}{\sigma_{K}^{2}\left(\tilde{P}\right)}\frac{\log(n)}{\rho n})$, respectively, and we see that ONA yields a stable and consistent community detection for both the row and column nodes, since the error rates go to zero as n→∞ when $\tilde{P}$ is fixed. Especially, for the row nodes with mixed memberships, when the DCMM proposed in [9] reduces to MMSB and K=O(1), the error bound of the Mixed-SCORE in Theorem 2.2 of [9] is also $O(\frac{1}{\sigma_{K}\left(\tilde{P}\right)}\sqrt{\frac{\log(n)}{n}})$, which guarantees the theoretical optimality of our analysis for the row nodes. For the column nodes, when every column community enjoys similar sizes and K=O(1), our bound $O(\frac{1}{\sigma_{K}^{2}\left(\tilde{P}\right)}\frac{\log(n)}{\rho n})$ matches Corollary 3.2 in [13] up to a logarithmic factor, which guarantees the theoretical optimality of our analysis for column nodes. Furthermore, the optimality of our requirement on network sparsity and the theoretical upper bounds of ONA’s error rates is also supported by using the separation condition and sharp threshold criterion developed in [28].

## 3. The Overlapping and Degree-Corrected Non-Overlapping Model

In this section, we propose an extension of ONM by considering the degree heterogeneity, and we build theoretical guarantees for algorithm fitting our model.

Let $\theta_{c}$ be an $n_{c}\times 1$ vector whose $i_{c}$-th entry is the degree heterogeneity of column node $i_{c}$, for $i_{c}\in \left[n_{c}\right]$. Let $\Theta_{c}$ be an $n_{c}\times n_{c}$ diagonal matrix whose $i_{c}$-th diagonal element is $\theta_{c}\left(i_{c}\right)$. For $i_{r}\in \left[n_{r}\right],i_{c}\in \left[n_{c}\right]$, the extended model for generating *A* is:

(6) $$\begin{array}{c} \Omega :=\Pi_{r}P\Pi_{c}^{\prime }\Theta_{c},\;\;\;A\left(i_{r},i_{c}\right)∼\mathrm{Bernoulli}\left(\Omega \left(i_{r},i_{c}\right)\right). \end{array}$$

**Definition** **2.** *Call model (1)–(4), (6) the Overlapping and Degree-Corrected Non-overlapping model (ODCNM), and denote it by $ODCNM_{n_{r},n_{c}}\left(K_{r},K_{c},P,\Pi_{r},\Pi_{c},\Theta_{c}\right)$.*

Note that, under ODCNM, the maximum element of *P* can be larger than 1, since $\max_{i_{c}\in \left[n_{c}\right]}\theta_{c}\left(i_{c}\right)$ also controls the sparsity of directed network N. The following proposition guarantees that ODCNM is identifiable in terms of $P,\Pi_{r}$, and $\Pi_{c}$, and such identifiability is similar to that of DCSBM.

**Proposition** **2.** *If conditions (I1) and (I2) hold, ODCNM is identifiable for the membership matrices: For eligible $\left(P,\Pi_{r},\Pi_{c},\Theta_{c}\right)$ and $\left(\overset{ˇ}{P},{\overset{ˇ}{\Pi }}_{r},{\overset{ˇ}{\Pi }}_{c},{\overset{ˇ}{\Theta }}_{c}\right)$, if $\Pi_{r}P\Pi_{c}^{\prime }\Theta_{c}={\overset{ˇ}{\Pi }}_{r}\overset{ˇ}{P}{\overset{ˇ}{\Pi }}_{c}^{\prime }{\overset{ˇ}{\Theta }}_{c}$, then $\Pi_{r}={\overset{ˇ}{\Pi }}_{r}$ and $\Pi_{c}={\overset{ˇ}{\Pi }}_{c}$.*

**Remark** **2.** *By setting $\theta_{c}\left(i_{c}\right)=\rho$ for $i_{c}\in \left[n_{c}\right]$, ODCNM reduces to ONM, and this is the reason for why ODCNM can be seen as an extension of ONM. Meanwhile, though DCScBM [18] can model directed networks with degree heterogeneities for both row and column nodes, DCScBM does not allow the overlapping property for row nodes. For comparison, our ODCNM allows row nodes to have an overlapping property at the cost of losing the degree heterogeneities and requiring $K_{r}\leq K_{c}$ for model identifiability.*

### 3.1. A Spectral Algorithm for Fitting ODCNM

We now discuss our intuition for the design of our algorithm to fit ODCNM. Without causing confusion, we also use $U_{r},U_{c},B_{r},B_{c},\delta_{c},Y_{r}$ under ODCNM. Let $U_{c,*}\in R^{n_{c}\times K_{r}}$ be the row-normalized version of $U_{c}$, such that $U_{c,*}\left(i_{c},:\right)=\frac{U_{c}\left(i_{c},:\right)}{∥U_{c}\left(i_{c},:\right){∥}_{F}}$ for $i_{c}\in \left[n_{c}\right]$. Then, clustering the rows of $U_{c,*}$ using the k-means algorithm can return perfect clustering for column nodes, and this is guaranteed by the following lemma.

**Lemma** **2.** *Under $ODCNM_{n_{r},n_{c}}\left(K_{r},K_{c},P,\Pi_{r},\Pi_{c},\Theta_{c}\right)$, there exists a unique $K_{r}\times K_{r}$ matrix $B_{r}$ and a unique $K_{c}\times K_{r}$ matrix $B_{c}$, such that*

- *$U_{r}=\Pi_{r}B_{r}$, where $B_{r}=U_{r}\left(I_{r},:\right)$. Meanwhile, $U_{r}\left(i_{r},:\right)=U_{r}\left({\bar{i}}_{r},:\right)$ when $\Pi_{r}\left(i_{r},:\right)=\Pi_{r}\left({\bar{i}}_{r},:\right)$ for $i_{r},{\bar{i}}_{r}\in \left[n_{r}\right]$.*
- *$U_{c,*}=\Pi_{c}B_{c}$. Meanwhile, $U_{c,*}\left(i_{c},:\right)=U_{c,*}\left({\bar{i}}_{c},:\right)$ when $\ell \left(i_{c}\right)=\ell \left({\bar{i}}_{c}\right)$ for $i_{c},{\bar{i}}_{c}\in \left[n_{c}\right]$. Furthermore, when $K_{r}=K_{c}=K$, we have $∥B_{c}\left(k,:\right)-B_{c}{\left(l,:\right)∥}_{F}=\sqrt{2}$ for all 1≤k<l≤K.*

Recall that we set $\delta_{c}=\min_{k\neq l}{∥B_{c}\left(k,:\right)-B_{c}\left(l,:\right)∥}_{F}$ by Lemma 2; $\delta_{c}=\sqrt{2}$ when $K_{r}=K_{c}=K$ under $ODCNM_{n_{r},n_{c}}\left(K_{r},K_{c},P,\Pi_{r},\Pi_{c},\Theta_{c}\right)$. However, when $K_{r}<K_{c}$, it is a challenge to obtain a positive lower bound of $\delta_{c}$; see the proof of Lemma 2 for details.

Under ODCNM, to recover $\Pi_{c}$ from $U_{c}$, since $U_{c,*}$ has $K_{c}$ distinct rows, applying the k-means algorithm on all rows of $U_{c,*}$ returns true column communities by Lemma 2. To recover $\Pi_{r}$ from $U_{r}$, the same idea as that of under ONM can be followed.

Based on the above analysis, we are now ready to present the following algorithm, which we call Ideal ODCNA. Input $\Omega ,K_{r},K_{c}$ with $K_{r}\leq K_{c}$. Output: $\Pi_{r}$ and *ℓ*.

- Let $\Omega =U_{r}\Lambda U_{c}^{\prime }$ be the compact SVD of Ω, such that $U_{r}\in R^{n_{r}\times K_{r}},U_{c}\in R^{n_{c}\times K_{r}},\Lambda \in R^{K_{r}\times K_{r}},U_{r}^{\prime }U_{r}=I_{K_{r}},U_{c}^{\prime }U_{c}=I_{K_{r}}$. Let $U_{c,*}$ be the row-normalization of $U_{c}$.
- For row nodes, they are the same as that of Ideal ONA.
  For column nodes: run k-means on $U_{c,*}$ assuming there are $K_{c}$ column communities to obtain *ℓ*.

We now extend the ideal case to the real case. Let ${\hat{U}}_{c,*}\in R^{n_{c}\times K_{r}}$ be the row-normalized version of ${\hat{U}}_{c}$, such that ${\hat{U}}_{c,*}\left(i_{c},:\right)=\frac{{\hat{U}}_{c}\left(i_{c},:\right)}{∥{\hat{U}}_{c}\left(i_{c},:\right){∥}_{F}}$ for $i_{c}\in \left[n_{c}\right]$. The Overlapping and Degree-Corrected Non-overlapping Algorithm (ODCNA for short) is a natural extension of the Ideal ODCNA to the real case, where all steps of ODCNA are the same as ONA except for those for column nodes. ODCNA applies k-means on ${\hat{U}}_{c,*}$ to obtain $\hat{\ell }$.

### 3.2. Main Results for ODCNA

Set $\theta_{c,\max}=\max_{i_{c}\in \left[n_{c}\right]}\theta_{c}\left(i_{c}\right),\theta_{c,\min}=\min_{i_{c}\in \left[n_{c}\right]}\theta_{c}\left(i_{c}\right)$, and $P_{\max}=\max_{k\in \left[K_{r}\right],l\in \left[n_{c}\right]}P\left(k,l\right)$. Assume that

**Assumption** **2.** *$P_{\max}\max(\theta_{c,\max}n_{r},∥\theta_{c}{∥}_{1})\geq \log\left(n_{r}+n_{c}\right)$.*

The next theorem is the main theoretical result for ODCNA, where we also use the same measurements as ONA to measure the performances of ODCNA.

**Theorem** **2.** *Under $ODCNM_{n_{r},n_{c}}\left(K_{r},K_{c},P,\Pi_{r},\Pi_{c},\Theta_{c}\right)$, when Assumption 2 holds, suppose $\sigma_{K_{r}}\left(\Omega \right)\geq C\sqrt{\theta_{c,\max}\left(n_{r}+n_{c}\right)\log\left(n_{r}+n_{c}\right)}$, with a probability at least $1-o({\left(n_{r}+n_{c}\right)}^{-\alpha })$,*

- *For the row nodes,*
$$\begin{array}{cc} \; & \max_{i_{r}\in \left[n_{r}\right]}{∥e_{i_{r}}^{\prime }\left({\hat{\Pi }}_{r}-\Pi_{r}P_{r}\right)∥}_{1}=O\left(\varpi \kappa \left(\Pi_{r}^{\prime }\Pi_{r}\right)K_{r}\sqrt{\lambda_{1}\left(\Pi_{r}^{\prime }\Pi_{r}\right)}\right). \end{array}$$
- *For the column nodes,*
$$\begin{array}{c} {\hat{f}}_{c}=O\left(\frac{\theta_{c,\max}^{2}K_{r}K_{c}\max(\theta_{c,\max}n_{r},∥\theta_{c}{∥}_{1})n_{c,\max}\log\left(n_{r}+n_{c}\right)}{\sigma_{K_{r}}^{2}\left(P\right)\theta_{c,\min}^{4}\delta_{c}^{2}m_{V_{c}}^{2}\sigma_{K_{r}}^{2}\left(\Pi_{r}\right)n_{c,K_{r}}n_{c,\min}}\right), \end{array}$$
  *where $m_{V_{c}}$ is a parameter defined in the proof of this theorem, and it is 1 when $K_{r}=K_{c}$. Especially, when $K_{r}=K_{c}=K$,*
$$\begin{array}{c} {\hat{f}}_{c}=O\left(\frac{\theta_{c,\max}^{2}K^{2}\max(\theta_{c,\max}n_{r},∥\theta_{c}{∥}_{1})n_{c,\max}\log\left(n_{r}+n_{c}\right)}{\sigma_{K}^{2}\left(P\right)\theta_{c,\min}^{4}\sigma_{K}^{2}\left(\Pi_{r}\right)n_{c,\min}^{2}}\right). \end{array}$$

Adding some conditions on model parameters, we have the following corollary.

**Corollary** **2.** *Under $ODCNM_{n_{r},n_{c}}\left(K_{r},K_{c},P,\Pi_{r},\Pi_{c},\Theta_{c}\right)$, suppose that conditions in Theorem 2 hold, and further, suppose that $\lambda_{K_{r}}\left(\Pi_{r}^{\prime }\Pi_{r}\right)=O\left(\frac{n_{r}}{K_{r}}\right),n_{c,\min}=O\left(\frac{n_{c}}{K_{c}}\right)$, with a probability of at least $1-o({\left(n_{r}+n_{c}\right)}^{-\alpha })$,*

- *For row nodes, when $K_{r}=K_{c}=K$,*
  $$\begin{array}{cc} \; & \max_{i_{r}\in \left[n_{r}\right]}{∥e_{i_{r}}^{\prime }\left({\hat{\Pi }}_{r}-\Pi_{r}P_{r}\right)∥}_{1}=O\left(\frac{K^{2}\sqrt{\theta_{c,\max}}\left(\sqrt{\frac{C\max(n_{r},n_{c})}{\min(n_{r},n_{c})}}+\sqrt{\log(n_{r}+n_{c})}\right)}{\theta_{c,\min}\sigma_{K}\left(P\right)\sqrt{n_{c}}}\right). \end{array}$$
- *For column nodes, ${\hat{f}}_{c}=O\left(\frac{\theta_{c,\max}^{2}K_{r}^{2}K_{c}^{2}\max(\theta_{c,\max}n_{r},∥\theta_{c}{∥}_{1})\log\left(n_{r}+n_{c}\right)}{\sigma_{K_{r}}^{2}\left(P\right)\theta_{c,\min}^{4}\delta_{c}^{2}m_{V_{c}}^{2}n_{r}n_{c}}\right)$. When $K_{r}=K_{c}=K$,*
  $$\begin{array}{c} {\hat{f}}_{c}=O\left(\frac{\theta_{c,\max}^{2}K^{4}\max(\theta_{c,\max}n_{r},∥\theta_{c}{∥}_{1})\log\left(n_{r}+n_{c}\right)}{\sigma_{K}^{2}\left(P\right)\theta_{c,\min}^{4}n_{r}n_{c}}\right). \end{array}$$

*Especially, when*$n_{r}=O\left(n\right),n_{c}=O\left(n\right),K_{r}=O\left(1\right)$*and*$K_{c}=O\left(1\right)$,

- *For row nodes, when $K_{r}=K_{c}$,*
  $$\begin{array}{c} \max_{i_{r}\in \left[n_{r}\right]}{∥e_{i_{r}}^{\prime }\left({\hat{\Pi }}_{r}-\Pi_{r}P_{r}\right)∥}_{1}=O\left(\frac{\sqrt{\theta_{c,\max}\log\left(n\right)}}{\theta_{c,\min}\sigma_{K}\left(P\right)\sqrt{n}}\right). \end{array}$$
- *For column nodes,${\hat{f}}_{c}=O\left(\frac{\theta_{c,\max}^{2}\max(\theta_{c,\max}n_{r},∥\theta_{c}{∥}_{1})\log\left(n\right)}{\sigma_{K_{r}}^{2}\left(P\right)\theta_{c,\min}^{4}\delta_{c}^{2}m_{V_{c}}^{2}n^{2}}\right)$. When $K_{r}=K_{c}=K$,*
  $$\begin{array}{c} {\hat{f}}_{c}=O\left(\frac{\theta_{c,\max}^{2}\max(\theta_{c,\max}n_{r},∥\theta_{c}{∥}_{1})\log\left(n\right)}{\sigma_{K}^{2}\left(P\right)\theta_{c,\min}^{4}n^{2}}\right). \end{array}$$

If we further set $\theta_{c,\max}=O\left(\rho \right)$ and $\theta_{c,\min}=O\left(\rho \right)$, we have the below corollary.

**Corollary** **3.** *Under $ODCNM_{n_{r},n_{c}}\left(K_{r},K_{c},P,\Pi_{r},\Pi_{c},\Theta_{c}\right)$, suppose that the conditions in Theorem 2 hold, and further, suppose that $\lambda_{K_{r}}\left(\Pi_{r}^{\prime }\Pi_{r}\right)=O\left(\frac{n_{r}}{K_{r}}\right),n_{c,\min}=O\left(\frac{n_{c}}{K_{c}}\right)$ and $\theta_{c,\max}=O\left(\rho \right),\theta_{c,\min}=O\left(\rho \right)$, with a probability of at least $1-o({\left(n_{r}+n_{c}\right)}^{-\alpha })$,*

- *For row nodes, when $K_{r}=K_{c}=K$,*
  $$\begin{array}{cc} \; & \max_{i_{r}\in \left[n_{r}\right]}{∥e_{i_{r}}^{\prime }\left({\hat{\Pi }}_{r}-\Pi_{r}P_{r}\right)∥}_{1}=O\left(\frac{K^{2}\left(\sqrt{\frac{C\max(n_{r},n_{c})}{\min(n_{r},n_{c})}}+\sqrt{\log(n_{r}+n_{c})}\right)}{\sigma_{K}\left(P\right)\sqrt{\rho n_{c}}}\right). \end{array}$$
- *For column nodes, ${\hat{f}}_{c}=O\left(\frac{K_{r}^{2}K_{c}^{2}\max\left(n_{r},n_{c}\right)\log\left(n_{r}+n_{c}\right)}{\sigma_{K_{r}}^{2}\left(P\right)\rho \delta_{c}^{2}m_{V_{c}}^{2}n_{r}n_{c}}\right)$. When $K_{r}=K_{c}=K$,*
  $$\begin{array}{c} {\hat{f}}_{c}=O\left(\frac{K^{4}\max\left(n_{r},n_{c}\right)\log\left(n_{r}+n_{c}\right)}{\sigma_{K}^{2}\left(P\right)\rho n_{r}n_{c}}\right). \end{array}$$

*Especially, when*$n_{r}=O\left(n\right),n_{c}=O\left(n\right),K_{r}=O\left(1\right)$*and*$K_{c}=O\left(1\right)$,

- *For row nodes, when $K_{r}=K_{c}$,*
  $$\begin{array}{c} \max_{i_{r}\in \left[n_{r}\right]}{∥e_{i_{r}}^{\prime }\left({\hat{\Pi }}_{r}-\Pi_{r}P_{r}\right)∥}_{1}=O\left(\frac{\sqrt{\log(n)}}{\sigma_{K}\left(P\right)\sqrt{\rho n}}\right). \end{array}$$
- *For column nodes,${\hat{f}}_{c}=O\left(\frac{\log(n)}{\sigma_{K_{r}}^{2}\left(P\right)\rho \delta_{c}^{2}m_{V_{c}}^{2}n}\right)$. When $K_{r}=K_{c}=K$,*
  $$\begin{array}{c} {\hat{f}}_{c}=O\left(\frac{\log(n)}{\sigma_{K}^{2}\left(P\right)\rho n}\right). \end{array}$$

By setting $\Theta_{c}=\rho I$, ODCNM degenerates to ONM. By comparing Corollaries 1 and 3, we see that theoretical results under ODCNM are consistent with those under ONM when ODCNM degenerates to ONM for the case where $K_{r}=K_{c}=K$.

## 4. Simulations

In this section, we present some simulations to investigate the performances of the two proposed algorithms. We measure their performances using the Mixed-Hamming error rate (MHamm for short) for row nodes, and the Hamming error rate (Hamm for short) for the column nodes defined below

$$\begin{array}{cc} \; & \mathrm{MHamm}=\frac{\min_{\pi \in S_{K_{r}}}{∥{\hat{\Pi }}_{r}\pi -\Pi_{r}∥}_{1}}{n_{r}},\mathrm{Hamm}=\frac{\min_{\pi \in S_{K_{c}}}{∥{\hat{\Pi }}_{c}\pi -\Pi_{c}∥}_{0}}{n_{c}}, \end{array}$$

where $S_{K_{r}}$ is the set of all permutations of $\left\{1,2,\ldots ,K_{r}\right\}$, $S_{K_{c}}$ is the set of all permutations of $\left\{1,2,\ldots ,K_{c}\right\}$; ${\hat{\Pi }}_{c}\in R^{n_{c}\times K_{c}}$ is defined as ${\hat{\Pi }}_{c}\left(i_{c},k\right)=1$ if $\hat{\ell }\left(i_{c}\right)=k$, and 0 otherwise for $i_{c}\in \left[n_{c}\right],k\in \left[K_{c}\right]$.

For all simulations in this section, the parameters $\left(n_{r},n_{c},K_{r},K_{c},P,\rho ,\Pi_{r},\Pi_{c},\Theta_{c}\right)$ are set as below. Unless specified, set $n_{r}=400,n_{c}=300,K_{r}=3,K_{c}=4$. For the column nodes, generate $\Pi_{c}$ by setting each column node belonging to one of the column communities with equal probability. Let each row community have 100 pure nodes, and let all the mixed row nodes have memberships (0.6,0.3,0.1). $P=\rho \tilde{P}$ is set independently under ONM and ODCNM. Under ONM, ρ is 0.5 in Experiment 1, and we study the influence of ρ in Experiment 2. Under ODCNM, for $z_{c}\geq 1$, we generate the degree parameters for the column nodes as below: let $\theta_{c}\in R^{n_{c}\times 1}$, such that $1/\theta_{c}\left(i_{c}\right)\overset{iid}{∼}U\left(1,z_{c}\right)$ for $i_{c}\in \left[n_{c}\right]$, where $U(1,z_{c})$ denotes the uniform distribution on $\left[1,z_{c}\right]$. We study the influences of $z_{c}$ and ρ under ODCNM in Experiments 3 and 4, respectively. For all settings, we report the averaged MHamm and the averaged Hamm over 50 repetitions.

**Experiment 1: Changing $n_{c}$ under ONM.** Let $n_{c}$ range over {50,100,150,…,300}. For this experiment, *P* is set as

$$P=\rho \left[\begin{array}{cccc} 1 & 0.3 & 0.2 & 0.3\\ 0.2 & 0.9 & 0.1 & 0.2\\ 0.3 & 0.2 & 0.8 & 0.3 \end{array}\right].$$

**Figure 1:**
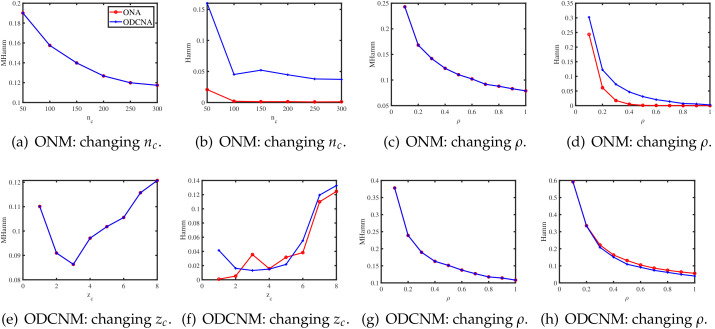
Estimation errors of ONA and ODCNA.

Let ρ=0.5 for this experiment designed under ONM. The numerical results are shown in panels (a) and (b) of Figure 1. The results show that as $n_{c}$ increases, ONA and ODCNA perform better. For the row nodes, since both ONA and ODCNA apply the SP algorithm on $\hat{U}$ to estimate $\Pi_{r}$, the estimated row membership matrices of ONA and ODCNA are same, and hence, MHamm for ONA is always equal to that of ODCNA.

**Experiment 2: Changing ρ under ONM.***P* is set the same as in Experiment 1, and we let the range of ρ be {0.1,0.2,…,1} to study the influence of ρ on the performances of ONA and ODCNA under ONM. The results are displayed in panels (c) and (d) of Figure 1. From the results, we can see that both methods perform better as ρ increases, since a larger ρ gives more edges generated in a directed network.

**Experiment 3: Change $z_{c}$ under ODCNM.***P* is set to be the same as Experiment 1, and ρ=0.5. Let $z_{c}$ range in {1,2,…,8}. Increasing $z_{c}$ decreases the edges generated under ODCNM. Panels (e) and (f) in Figure 1 display the simulation results of this experiment. The results show that generally, increasing the variability of the node degrees makes it harder to detect the node memberships for both ONA and ODCNA. Though ODCNA is designed under ODCNM, it holds similar performances as ONA for directed networks in which column nodes have various degrees in this experiment, and this is consistent with our theoretical findings in Corollaries 1 and 2.

**Experiment 4: Change ρ under ODCNM.** Setting $z_{c}=3$, *P* is set to be the same as in Experiment 1, and let ρ range in {0.1,0.2,…,1} under ODCNM. Panels (g) and (h) in Figure 1 display the simulation results of this experiment. The performances of the two proposed methods are similar as those of Experiment 2.

**Remark** **3.** *For visuality, we plot A generated under ONM. Let $n_{r}=24,n_{c}=20,K_{r}=2,K_{c}=2$, and*

$$P=\left[\begin{array}{ccc} 1 & 0.2 & \\ 0.1 & 0.9 \end{array}\right].$$

**Figure 2:**
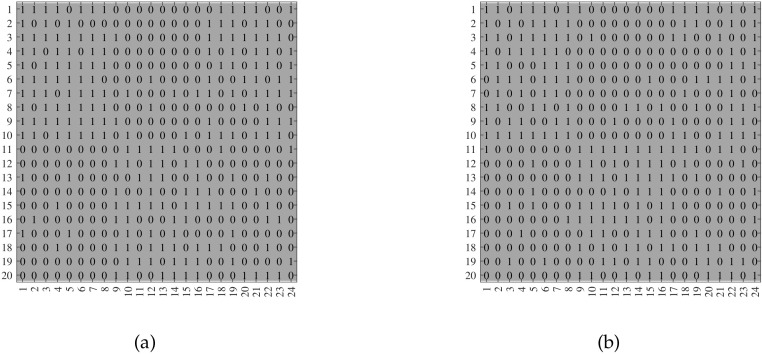
For adjacency matrix in panel (**a**), MHamm and Hamm for ONA are 0.0544 and 0, respectively. For adjacency matrix in panel (**b**), MHamm and Hamm for ONA are 0.1004 and 0, respectively. ODCNA enjoys same error rates as ONA. x-axis: row nodes; y-axis: column nodes.

*For the row nodes, let $\Pi_{r}\left(i_{r},1\right)=1$ for $1\leq i_{r}\leq 8$, $\Pi_{r}\left(i_{r},2\right)=1$ for $9\leq i_{r}\leq 16$, and $\Pi_{r}\left(r_{r},:\right)=\left[0.7\;\;0.3\right]$ for $17\leq i_{r}\leq 24$. For the column nodes, let $\ell (i_{c})=1$ for $1\leq i_{c}\leq 10$, and $\ell (i_{c})=2$ for $11\leq i_{c}\leq 20$. For the above setting, we generate two random adjacency matrices in Figure 2, where we also report the error rates of ONA and ODCNA. Note that, since the adjacency matrices are shown in Figure 2, and as $\Pi_{r},\ell ,K_{r}$, and $K_{c}$ are known here, readers can apply ONA and ODCNA to A in Figure 2 to check the effectiveness of ONA and ODCNA.*

**Remark** **4.** *For visuality, we also plot a directed network as well as its adjacency matrix generated under ONM. Let $n_{r}=30,n_{c}=30,K_{r}=2,K_{c}=3$, and*

$$P=0.9\left[\begin{array}{ccc} 1 & 0.1 & 0.1\\ 0.1 & 0.9 & 0.1 \end{array}\right].$$

**Figure 3:**
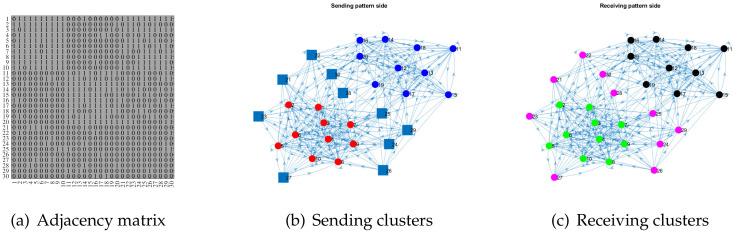
Illustration of a simulated directed network generated under ONM. Panels (**a**–**c**) show the adjacency matrix, the sending clusters, and the receiving clusters of this simulated directed network, respectively. For this directed network, MHamm and Hamm for ONA (and ODCNA) are 0.0615 (0.0615) and 0 (0.2333), respectively. In panels (**b**,**c**), the dots in the same color are pure nodes in the same sending (receiving) clusters, and the square indicates the mixed nodes with weight 0.7 belonging to red sending clusters, and weight 0.3 belonging to blue sending clusters, where the sending and receiving clusters are obtained by $\Pi_{r}$ and *ℓ* provided in Remark 4.

*For row nodes, let $\Pi_{r}\left(i_{r},1\right)=1$ for $1\leq i_{r}\leq 10$, $\Pi_{r}\left(i_{r},2\right)=1$ for $11\leq i_{r}\leq 20$, and $\Pi_{r}\left(i_{r},:\right)=\left[0.7\;\;0.3\right]$ for $21\leq i_{r}\leq 30$. For column nodes, let $\ell (i_{c})=1$ for $1\leq i_{c}\leq 10$, $\ell (i_{c})=2$ for $11\leq i_{c}\leq 20$, and $\ell (i_{c})=3$ for $21\leq i_{c}\leq 30$. For the above setting, we generate one adjacency matrix in panel (a) of Figure 3, where we also report the error rates of ONA and ODCNA. Furthermore, panels (b) and (c) of Figure 3 show the sending pattern and receiving pattern sides of this simulated directed network, respectively.*

## 5. Real Data Analysis

For real-world directed networks, since nodes always have various degrees, we only apply ODCNA to deal with real-world datasets in this section. For the real-world directed networks analyzed in this section, the row nodes are always same as the column nodes, so we do not use subscript *r* and *c* to distinguish the row and column nodes here, and we let $n_{r}=n_{c}=n$. Meanwhile, the number of row communities is equal to that of the column communities; i.e, $K_{r}=K_{c}=K$ for real data, where we always set $K_{r}=K_{c}=K$, as analyzed in [18], since it is a challenge to determine the number of row (column) communities for real-world directed networks without prior knowledge. When the row nodes are the same as the column nodes, A(i,j)=1 means that a directed edge is sent from node *i* to node *j*. Thus, for any node, it has two patterns, the sending pattern and the receiving pattern. For the sending (receiving) pattern, we use the sending (receiving) cluster to denote the prior row (column) community, where we use the sending and receiving patterns to distinguish the behaviors of any node having the two patterns, as was performed in [18].

For ${\hat{\Pi }}_{r}$ obtained from ODCNA, we call node *i* a highly mixed node if $0.8\geq \max_{1\leq k\leq K}{\hat{\Pi }}_{r}\left(i,k\right)$, where 0.8 is a threshold. Here, 0.8 is a moderate value to define highly mixed nodes, and we can also choose 0.9, 0.95, or some other values in (0,1). However, we choose 0.8 as the threshold, because setting the threshold to be larger (or lesser) than 0.8 may be too restrictive (loose) to define highly mixed nodes. The definition of highly mixed node is important, since it tells us whether a node only belongs to one community or belongs to multiple communities. Let τ be the proportion of highly mixed row nodes among all nodes, to measure the mixability of a directed network, i.e, $\tau =\frac{\left|i\in [n]:i\;\mathrm{is}\;a\;\mathrm{highly}\;\mathrm{mixed}\;\mathrm{node}\right|}{n}$. Meanwhile, we let ${\hat{\ell }}_{r}$ be an n×1 vector, such that ${\hat{\ell }}_{r}\left(i\right)={\mathrm{argmax}}_{1\leq k\leq K}{\hat{\Pi }}_{r}\left(i,k\right)$, where we use ${\hat{\ell }}_{r}\left(i\right)$ to denote the home base sending pattern cluster of node *i*. Set

$$\begin{array}{c} {\mathrm{Hamm}}_{rc}=\frac{\min_{\pi \in S_{K}}{∥{\hat{\Pi }}_{c}\pi -{\tilde{\hat{\Pi }}}_{r}∥}_{0}}{n}, \end{array}$$

where $S_{K}$ is the set of all permutations of {1,2,…,K}; ${\tilde{\hat{\Pi }}}_{r}\in R^{n\times K}$ is defined as ${\tilde{\hat{\Pi }}}_{r}\left(i,k\right)=1$ if ${\hat{\ell }}_{r}\left(i\right)=k$, and 0 otherwise for i∈[n],k∈[K]. ${\mathrm{Hamm}}_{rc}$ is defined to measure the difference between the sending and receiving pattern clusters. After defining τ and ${\mathrm{Hamm}}_{rc}$, we see that a larger τ indicates a directed network in which a large proposition of nodes are highly mixed nodes with a sending pattern, and a larger ${\mathrm{Hamm}}_{rc}$ indicates that the sending pattern differs a lot with the receiving pattern. For i∈[n], let $d_{sending}\left(i\right)=\sum_{j=1}^{n}A\left(i,j\right)$ denote the total number of edges sent by node *i*, and let $d_{receiving}\left(i\right)=\sum_{j=1}^{n}A\left(j,i\right)$ denote the total number of edges that are received by node *i*. Call $d_{sending}\left(i\right)$ and $d_{receiving}\left(i\right)$ the sending degree and receiving degree of node *i*, respectively. For real-world directed networks, we find that there are many nodes whose sending degree or receiving degree are zero, and so we need the following pre-processing steps before analyzing the real data:

(a) Set $S_{sending,0}=\left\{i\in \left[n\right]:d_{sending}\left(i\right)=0\right\}$ and $S_{receiving,0}=\left\{i\in \left[n\right]:d_{receiving}\left(i\right)=0\right\}$.
(b) Set $S_{degree,0}=S_{sending,0}⋃S_{receiving,0}$.
(c) Update *A* by removing the nodes in $S_{degree,0}$.
(d) Repeat (a)–(c) until $S_{degree,0}$ is a null set.
(e) After obtaining *A* through the above four steps, obtain the largest connected component of *A*.

**Figure 4:**
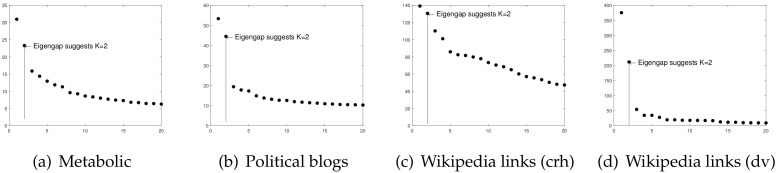
Leading 20 singular values of adjacency matrices for real-world directed networks used in this paper.

We now describe the real-world directed networks analyzed in this paper:

**Metabolic**: This is a directed network representing the metabolic reactions of E.coli bacteria. In this data, node means metabolite, and a directed edge from node *i* to node *j* means that there is a reaction where node *i* is an input and node *j* is a product [29]. These data can be downloaded from http://networksciencebook.com/translations/en/resources/data.html. The original dat has 1039 nodes; after preprocessing, $A\in {\left\{0,1\right\}}^{893\times 893}$. To estimate *K*, we plot the leading 20 singular values of *A*, and panel (a) of Figure 4 shows the result that suggests that K=2 for these data, where [18] also applies the idea of an eigengap to estimate *K* for real-world directed networks with an unknown number of communities.

**Political blogs**: this is a directed network of hyperlinks between weblogs on US politics [30], and it can be downloaded from http://www-personal.umich.edu/~mejn/netdata/. Political blogs send and receive hyperlinks to and from blogs for the same political persuasion [18], so node means blog and edge means hyperlink in these data. The original network has 1490 nodes. After removing nodes with zero degrees via pre-processing steps, there are 814 nodes left; i.e., $A\in {\left\{0,1\right\}}^{813\times 813}$ for these data. Since there are two parties, “liberal” and “conservative”, *K* is 2 for both the sending and receiving pattern communities for these data. [18] applies their DI-SIM algorithm to the Political blogs network, assuming that all nodes have non-overlapping property. In this paper, we apply our ODCNA algorithm on these data to study its asymmetric structure on the overlapping property.

**Wikipedia links (crh)**: This directed network represents the wikilinks of the Wikipedia website in the Crimean Turkish language (crh). Node means article, and the directed edge between two articles is the wikilink [31]. These data can be downloaded from http://konect.cc/networks/wikipedia_link_crh/. The original data have 8286 nodes. After pro-processing, $A\in {\left\{0,1\right\}}^{3555\times 3555}$. Panel (c) of Figure 4 suggests K=2 for this data.

**Wikipedia links (dv)**: These data represent the wikilinks of the Wikipedia website in the Divehi language (dv), where node means article and the directed edge is a wikilink [31]. These data can be downloaded from http://konect.cc/networks/wikipedia_link_dv/. The original data has 4266 nodes. After removing nodes with zero degrees, $A\in {\left\{0,1\right\}}^{2394\times 2394}$. Panel (d) of Figure 4 suggests K=2 for these data.

The proportion of highly mixed nodes and ${\mathrm{Hamm}}_{rc}$ for these directed networks are reported in Table 1 when assuming that nodes in sending (receiving) clusters having an overlapping (non-overlapping) property. For the Metabolic network, the results show that the sending pattern differs a lot with the receiving pattern, since ${\mathrm{Hamm}}_{rc}=0.2497$ is quite large, and there are 893×0.1209≈108 highly mixed nodes in the sending pattern. For the Political blogs network, there is a slight asymmetric structure between the sending pattern and the receiving pattern, since ${\mathrm{Hamm}}_{rc}=0.0443$ is small. Meanwhile, for the sending pattern of Political blogs, there are 813×0.0246≈20 highly mixed nodes. Thus, we may conclude that though there is a slight asymmetric structure in sending and receiving patterns for Political blogs, there are 20 highly mixed nodes in the sending pattern. For the Wikipedia links (crh), they have a slight asymmetric structure between sending and receiving patterns, and there are 3555×0.0444≈158 highly mixed nodes in the sending pattern. For the Wikipedia links (dv) network, it has a large number of highly mixed nodes for its large τ, and a heavy asymmetric structure in sending and receiving patterns for its large ${\mathrm{Hamm}}_{rc}$. Generally, Table 1 suggests that if there are a large number of highly mixed nodes in the sending pattern, there is a heavy asymmetric structure between the sending and receiving patterns, and vice versa.

**Figure 5:**
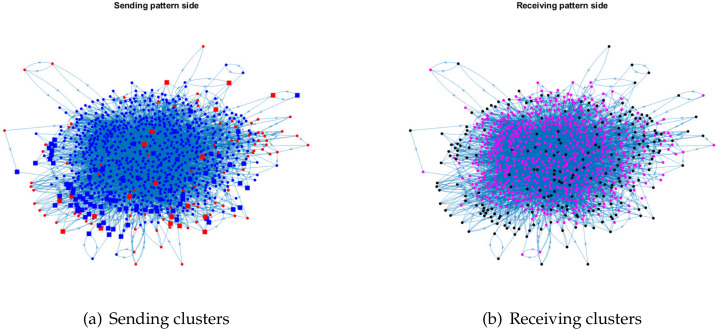
Sending and receiving clusters detected by ODCNA for Metabolic network when assuming that nodes in a sending (receiving) pattern have an overlapping (non-overlapping) property. Colors indicate clusters detected using ODCNA, and squares indicate highly mixed nodes, where sending clusters are obtained using ${\hat{\ell }}_{r}$, the home base sending pattern community, and receiving clusters are obtained by $\hat{\ell }$ from ODCNA.

**Figure 6:**
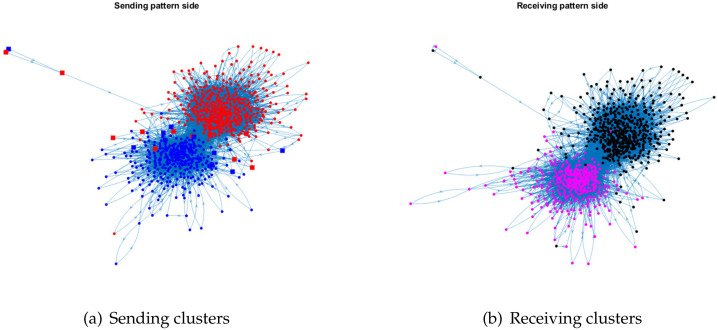
Sending and receiving clusters detected by ODCNA for Political blogs network. Colors indicate clusters and square indicates highly mixed nodes.

**Figure 7:**
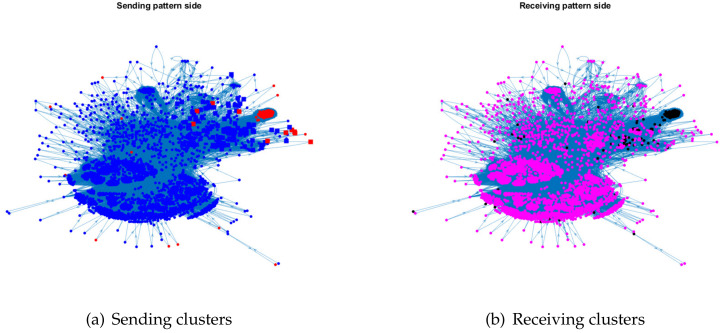
Sending and receiving clusters detected by ODCNA for Wikipedia links (crh) network. Colors indicate clusters and square indicates highly mixed nodes.

**Figure 8:**
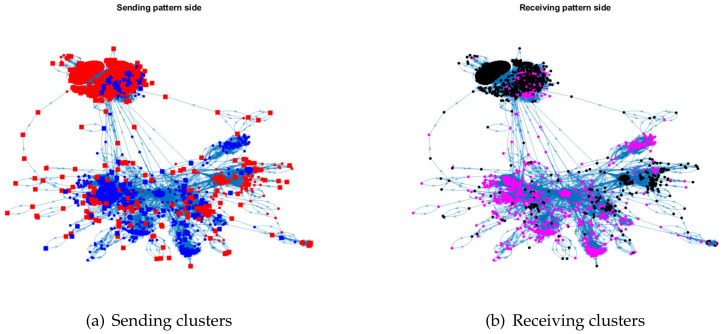
Sending and receiving clusters detected by ODCNA for Wikipedia links (dv) network. Colors indicate clusters and square indicates highly mixed nodes.

For visualization, we plot the sending clusters and receiving clusters detected by ODCNA for these directed networks when assuming that nodes in the sending (receiving) clusters have an overlapping (non-overlapping) property, i.e., when the input adjacency matrix of the ODCNA approach is *A*. The results are shown in Figure 5, Figure 6, Figure 7 and Figure 8, where we also show the highly mixed nodes in sending clusters detected by ODCNA. We see that there exists a clear asymmetric structure between the sending and receiving patterns for Metabolic and Wikipedia links (dv), as shown in Figure 5 and Figure 8, while there is a slight asymmetric structure between the sending and receiving patterns for Political blogs and Wikipedia links (crh), as shown in Figure 6 and Figure 7. Furthermore, most nodes are in the same sending (receiving) cluster for Metabolic and Wikipedia links (crh), while the two sending (receiving) clusters for Political blogs and Wikipedia links (crh) have close sizes. The results also show that most highly mixed nodes have many edges, while some highly mixed nodes have only a few edges, where such a phenomenon can be explained easily, since nodes with many edges tend to have an overlapping property, while it is difficult to detect a community for nodes with only a few edges, and ODCNA tends to treat such nodes as highly mixed nodes.

Furthermore, for real-world directed networks, since we have no prior knowledge on whether nodes in the sending pattern side or the receiving pattern side or both sides have overlapping property, simply inputting *A* with *K* sending (receiving) pattern communities in our ODCNA algorithm is not enough. To solve this problem, we also apply ODCNA on $A^{\prime }$, and the numerical results are provided in Table 2, where the results show that there also exist highly mixed nodes in the receiving pattern for these directed networks, and there also exists a heavy asymmetric structure between the sending and receiving clusters for the Metabolic and Wikipedia links (dv), while there also exists a slight asymmetric structure between the sending and receiving clusters for the Political blogs and Wikipedia links (crh).

## 6. Discussion

In this paper, we introduced Overlapping and Non-overlapping models and their extension, by considering the degree heterogeneity. The models can model a directed network with $K_{r}$ row communities and $K_{c}$ column communities, in which the row node can belong to multiple sending clusters, while the column node only belongs to one of the receiving clusters. The proposed models are identifiable under the case when $K_{r}\leq K_{c}$, and some other popular constraints on the connectivity matrix and membership matrices. For comparison, modeling a directed network in which the row nodes have overlapping property while column nodes do not, with $K_{r}>K_{c}$, is unidentifiable. Meanwhile, since previous works have found that modeling directed networks in which both row and column nodes have an overlapping property with $K_{r}\neq K_{c}$ is unidentifiable, our identifiable ONM and ODCNM supply a gap in modeling overlapping directed networks when $K_{r}\neq K_{c}$. These models provide exploratory tools for studying community structure in directed networks with one side overlapping while another side is non-overlapping. Two spectral algorithms are designed to fit ONM and ODCNM. We also showed an estimation consistency under mild conditions for our methods. Especially, when ODCNM reduces to ONM, our theoretical results under ODCNM are consistent with those under ONM. The numerical results for the simulated directed networks generated under ONM and ODCNM support our theoretical results, and the results for real-world directed networks reveal the existence of highly mixed nodes and an asymmetric structure between the sending and receiving clusters.

The models and algorithms introduced in this paper are useful tools for studying the asymmetric structure for directed networks, and we wish that they can be widely applied in network science. However, perhaps the main limitation of the models is that $K_{r}$ and $K_{c}$ in the directed network are assumed as givens, and such a limitation also holds for the spectral clustering algorithms developed under the ScBM and DCScBM studied in [18,19,20]. In most community problems, the number of row communities and the number of column communities are unknown; therefore, a complete calculation and theoretical study requires not only the algorithms and their theoretically consistent estimations described in this paper, but also a method for estimating $K_{r}$ and $K_{c}$. A possible solution to this problem may be a combination of algorithms developed in this paper and the modularity for the directed networks developed in [32]. Meanwhile, our idea can be extended in many ways. In this paper, we only consider modeling an un-weighted directed network, and it is possible to extend our work to a weighted directed network. Our algorithms are designed based on the adjacency matrix, and it is possible to design spectral algorithms to fit ONM and ODCNM by applying the regularized Laplace matrix used in [11,12]. When detecting large-scale directed networks, the random projection-based and the random sampling-based spectral clustering ideas in [33] may be applied to accelerate our algorithms. We leave the studies of these problems to our future work.

## Figures and Tables

**Table 1:** The proportion of highly mixed nodes and the asymmetric structure measured by ${\mathrm{Hamm}}_{rc}$ for real-world directed networks considered in this paper when ODCNA’s input adjacency matrix is *A*; i.e., the case when assuming that nodes in a sending (receiving) pattern have overlapping (non-overlapping) property.

Data	τ	${\mathrm{Hamm}}_{\mathrm{rc}}$
Metabolic	0.1209	0.2497
Political blogs	0.0246	0.0443
Wikipedia links (crh)	0.0444	0.0307
Wikipedia links (dv)	0.4089	0.1466

**Table 2:** The proportion of highly mixed nodes and the asymmetric structure measured by ${\mathrm{Hamm}}_{rc}$ for real-world directed networks considered in this paper when ODCNA’s input adjacency matrix is $A^{\prime }$, i.e., the case when assuming that nodes in sending (receiving) pattern have non-overlapping (overlapping) property.

Data	τ	${\mathrm{Hamm}}_{\mathrm{rc}}$
Metabolic	0.0594	0.2945
Political blogs	0.1365	0.0443
Wikipedia links (crh)	0.1308	0.0543
Wikipedia links (dv)	0.3492	0.2059

## Data Availability

The data that support the findings of this study are available within the article.

## Abbreviations

The following abbreviations are used in this manuscript:

SBM	Stochastic Blockmodel
DCSBM	Degree-Corrected Stochastic Blockmodel
MMSB	Mixed Membership Stochastic Blockmodel
DCMM	Degree-Corrected Mixed Membership model
OCCAM	Overlapping Continuous Community Assignment model
ScBM	Stochastic co-Blockmodel
DCScBM	Degree-Corrected Stochastic co-Blockmodel
DiMMSB	Directed Mixed Membership Stochastic Blockmodel
ONM	Overlapping and Non-overlapping model
ODCNM	Overlapping and Degree-Corrected Non-overlapping model
SP	successive projection algorithm
ONA	Overlapping and Non-overlapping algorithm
ODCNA	Overlapping and Degree-Corrected Non-overlapping Algorithm

## Appendix A. Successive Projection Algorithm

Algorithm A1 is the Successive Projection algorithm.

**Algorithm A1** **Successive Projection (SP)** [27]
**Require:** Near-separable matrix $Y_{sp}=S_{sp}M_{sp}+Z_{sp}\in R_{+}^{m\times n}$, where $S_{sp},M_{sp}$ should satisfy Assumption 1 [27], the number *r* of columns to be extracted. **Ensure:** Set of indices K such that Y(K,:)≈S (up to permutation) 1: Compute ${\hat{U}}_{r}\in R^{n_{r}\times K_{r}}$ and ${\hat{U}}_{c}\in R^{n_{c}\times K_{r}}$ from the top-$K_{r}$-dimensional SVD of *A*. 2: Let $R=Y_{sp},K=\left\{\right\},k=1$. 3: **While**R≠0 and k≤r **do** 4: $k_{*}={\mathrm{argmax}}_{k}{∥R\left(k,:\right)∥}_{F}$. 5: $u_{k}=R\left(k_{*},:\right)$. 6: $R\leftarrow (I-\frac{u_{k}u_{k}^{\prime }}{∥u_{k}{∥}_{F}^{2}})R$. 7: $K=K\cup \{k_{*}\}$. 8: k = k + 1. 9: **end while**

## Appendix B. Proofs under ONM

### Appendix B.1. Proof of Proposition 1

**Proof.** By Lemma 1, let $U_{r}\Lambda U_{c}^{\prime }$ be the compact SVD of Ω, such that $\Omega =U_{r}\Lambda U_{c}^{\prime }$; since $\Omega =\Pi_{r}P\Pi_{c}^{\prime }={\overset{ˇ}{\Pi }}_{r}\overset{ˇ}{P}{\overset{ˇ}{\Pi }}_{c}^{\prime }$, we have $\Omega \left(I_{r},I_{c}\right)=P=\overset{ˇ}{P}$, which gives $P=\overset{ˇ}{P}$. By Lemma 1, since $U_{r}=\Pi_{r}U_{r}\left(I_{r},:\right)={\overset{ˇ}{\Pi }}_{r}U_{r}\left(I_{r},:\right)$, we have $\Pi_{r}={\overset{ˇ}{\Pi }}_{r}$ where we have used the fact that the inverse of $U_{r}\left(I_{r},:\right)$ exists. Since $\Omega =\Pi_{r}P\Pi_{c}^{\prime }={\overset{ˇ}{\Pi }}_{r}\overset{ˇ}{P}{\overset{ˇ}{\Pi }}_{c}^{\prime }=\Pi_{r}P{\overset{ˇ}{\Pi }}_{c}^{\prime }$, we have $\Pi_{r}P\Pi_{c}^{\prime }=\Pi_{r}P{\overset{ˇ}{\Pi }}_{c}^{\prime }$. By Lemma 7 of [21], we have $P\Pi_{c}^{\prime }=P{\overset{ˇ}{\Pi }}_{c}^{\prime }$, i.e., $\Pi_{c}X={\overset{ˇ}{\Pi }}_{c}X$, where we set $X=P^{\prime }\in R^{K_{c}\times K_{r}}$. Let $\overset{ˇ}{\ell }$ be the $n_{c}\times 1$ vector of column nodes labels obtained from ${\overset{ˇ}{\Pi }}_{c}$. For $i_{c}\in \left[n_{c}\right],k\in \left[K_{r}\right]$, from $\Pi_{c}X={\overset{ˇ}{\Pi }}_{c}X$, we have $\left(\Pi_{c}X\right)\left(i_{c},k\right)=\Pi_{c}\left(i_{c},:\right)X\left(:,k\right)=X\left(\ell \left(i_{c}\right),k\right)=X\left(\overset{ˇ}{\ell }\left(i_{c}\right),k\right)$, which means that we must have $\ell \left(i_{c}\right)=\overset{ˇ}{\ell }\left(i_{c}\right)$ for all $i_{c}\in \left[n_{c}\right]$, i.e., $\ell =\overset{ˇ}{\ell }$ and $\Pi_{c}={\overset{ˇ}{\Pi }}_{c}$. Note that for the special case $K_{r}=K_{c}=K$, $\Pi_{c}={\overset{ˇ}{\Pi }}_{c}$ can be obtained easily: since $P\Pi_{c}^{\prime }=P{\overset{ˇ}{\Pi }}_{c}^{\prime }$ and $P\in R^{K\times K}$ is assumed to be full rank, we have $\Pi_{c}={\overset{ˇ}{\Pi }}_{c}$. Thus, the proposition holds. □

### Appendix B.2. Proof of Lemma 1

**Proof.** For $U_{r}$, since $\Omega =U_{r}\Lambda U_{c}^{\prime }$ and $U_{c}^{\prime }U_{c}=I_{K_{r}}$, we have $U_{r}=\Omega U_{c}\Lambda^{-1}$. Recall that $\Omega =\Pi_{r}P\Pi_{c}^{\prime }$; we have $U_{r}=\Pi_{r}P\Pi_{c}^{\prime }U_{c}\Lambda^{-1}=\Pi_{r}B_{r}$, where we set $B_{r}=P\Pi_{c}^{\prime }U_{c}\Lambda^{-1}$. Since $U_{r}\left(I_{r},:\right)=\Pi_{r}\left(I_{r},:\right)B_{r}=B_{r}$, we have $B_{r}=U_{r}\left(I_{r},:\right)$. For $i_{r}\in \left[n_{r}\right]$, $U_{r}\left(i_{r},:\right)=e_{i_{r}}^{\prime }\Pi_{r}B_{r}=\Pi_{r}\left(i_{r},:\right)B_{r}$, so we have $U_{r}\left(i_{r},:\right)=U_{r}\left({\bar{i}}_{r},:\right)$ when $\Pi_{r}\left(i_{r},:\right)=\Pi_{r}\left({\bar{i}}_{r},:\right)$. For $U_{c}$, following a similar analysis as for $U_{r}$, we have $U_{c}=\Pi_{c}B_{c}$, where $B_{c}=P^{\prime }\Pi_{r}^{\prime }U_{r}\Lambda^{-1}$. Note that $B_{c}\in R^{K_{c}*K_{r}}$. Sure, $U_{c}\left(i_{c},:\right)=U_{c}\left({\bar{i}}_{c},:\right)$ when $\ell \left(i_{c}\right)=\ell \left({\bar{i}}_{c}\right)$ for $i_{c},{\bar{i}}_{c}\in \left[n_{c}\right]$. Now, we focus on the case where $K_{r}=K_{c}=K$. For this case, since $B_{c}\in R^{K_{c}*K_{r}}$, $B_{c}$ is full rank when $K_{r}=K_{c}$. Since $I_{K_{r}}=I_{K}=U_{c}^{\prime }U_{c}=B_{c}^{\prime }\Pi_{c}^{\prime }\Pi_{c}B_{c}$, we have $\Pi_{c}^{\prime }\Pi_{c}={\left(B_{c}B_{c}^{\prime }\right)}^{-1}$. Since $\Pi_{c}^{\prime }\Pi_{c}=\mathrm{diag}\left(n_{c,1},n_{c,2},\ldots ,n_{c,K}\right)$, we have $B_{c}B_{c}^{\prime }=\mathrm{diag}\left(\frac{1}{n_{c,1}},\frac{1}{n_{c,2}},\ldots ,\frac{1}{n_{c,K}}\right)$. When $K_{r}=K_{c}=K$, we have $B_{c}\left(k,:\right)B_{c}^{\prime }\left(l,:\right)=0$ for any k≠l and k,l∈[K]. Then, we have $B_{c}B_{c}^{\prime }=\mathrm{diag}(∥B_{c}{\left(1,:\right)∥}_{F}^{2},∥B_{c}{\left(2,:\right)∥}_{F}^{2},\ldots ,∥B_{c}\left(K,:\right){∥}_{F}^{2})=\mathrm{diag}\left(\frac{1}{n_{c,1}},\frac{1}{n_{c,2}},\ldots ,\frac{1}{n_{c,K}}\right)$, and the lemma follows. Note that when $K_{r}<K_{c}$, since $B_{c}$ is not full rank now, we cannot obtain $\Pi_{c}^{\prime }\Pi_{c}={\left(B_{c}B_{c}^{\prime }\right)}^{-1}$ from $I_{K_{r}}=B_{c}^{\prime }\Pi_{c}^{\prime }\Pi_{c}B_{c}$. Therefore, when $K_{r}<K_{c}$, the equality $∥B_{c}\left(k,:\right)-B_{c}{\left(l,:\right)∥}_{F}=\sqrt{\frac{1}{n_{c,k}}+\frac{1}{n_{c,l}}}$ does not hold for any k≠l. Additionally, we can only know that $U_{c}$ has $K_{c}$ distinct rows when $K_{r}<K_{c}$, but have no knowledge about the minimum distance between any two distinct rows of $U_{c}$. □

### Appendix B.3. Proof of Theorem 1

**Proof.** First, by Lemma 4 of [21], we have the below lemma.

**Lemma** **A1.**
*(Row-wise singular eigenvector error) Under $ONM_{n_{r},n_{c}}\left(K_{r},K_{c},P,\Pi_{r},\Pi_{c}\right)$, when Assumption 1 holds, suppose $\sigma_{K_{r}}\left(\Omega \right)\geq C\sqrt{\rho \left(n_{r}+n_{c}\right)\log\left(n_{r}+n_{c}\right)}$, with a probability of at least $1-o({\left(n_{r}+n_{c}\right)}^{-\alpha })$,*

$$\begin{array}{cc} \; & ∥{\hat{U}}_{r}{\hat{U}}_{r}^{\prime }-U_{r}U_{r}^{\prime }{∥}_{2\to \infty }=O\left(\frac{\sqrt{K_{r}}\left(\kappa \left(\Omega \right)\sqrt{\frac{\max(n_{r},n_{c})\mu }{\min(n_{r},n_{c})}}+\sqrt{\log(n_{r}+n_{c})}\right)}{\sqrt{\rho }\sigma_{K_{r}}\left(\tilde{P}\right)\sigma_{K_{r}}\left(\Pi_{r}\right)\sqrt{n_{c,K_{r}}}}\right), \end{array}$$

*where μ is the incoherence parameter defined as $\mu =\max(\frac{n_{r}{∥U_{r}∥}_{2\to \infty }^{2}}{K_{r}},\frac{n_{c}{∥U_{c}∥}_{2\to \infty }^{2}}{K_{r}})$.*

For the row nodes, when conditions in Lemma A1 hold, by Theorem 2 of [21], with a probability of at least $1-o({\left(n_{r}+n_{c}\right)}^{-\alpha })$ for any α>0, there exists a permutation matrix $P_{r}$ such that, for $i_{r}\in \left[n_{r}\right]$, we have

$$\begin{array}{c} ∥e_{i_{r}}^{\prime }\left({\hat{\Pi }}_{r}-\Pi_{r}P_{r}\right){∥}_{1}=O\left(\varpi \kappa \left(\Pi_{r}^{\prime }\Pi_{r}\right)K_{r}\sqrt{\lambda_{1}\left(\Pi_{r}^{\prime }\Pi_{r}\right)}\right). \end{array}$$

Next, we focus on the column nodes. By the Proof of Lemma 3 in [19], there exists an orthogonal matrix $\hat{O}$ such that

(A1) $$\begin{array}{c} ∥{\hat{U}}_{c}\hat{O}-U_{c}{∥}_{F}\leq \frac{2\sqrt{2K_{r}}∥A-\Omega ∥}{\sqrt{\lambda_{K_{r}}\left(\Omega^{\prime }\Omega \right)}}. \end{array}$$

Under $ONM_{n_{r},n_{c}}\left(K_{r},K_{c},P,\Pi_{r},\Pi_{c}\right)$, by Lemma 10 of [21], we have

(A2) $$\begin{array}{c} \sqrt{\lambda_{K_{r}}\left(\Omega^{\prime }\Omega \right)}\geq \rho \sigma_{K_{r}}\left(\tilde{P}\right)\sigma_{K_{r}}\left(\Pi_{r}\right)\sigma_{K_{r}}\left(\Pi_{c}\right). \end{array}$$

Since all column nodes are pure, $\sigma_{K_{r}}\left(\Pi_{c}\right)=\sqrt{n_{c,K_{r}}}$. By Lemma 3 of [21], when Assumption 1 holds with a probability at least $1-o({\left(n_{r}+n_{c}\right)}^{-\alpha })$, we have

(A3) $$\begin{array}{c} ∥A-\Omega ∥=O(\sqrt{\rho \max\left(n_{r},n_{c}\right)\log\left(n_{r}+n_{c}\right)}). \end{array}$$

Substituting the two bounds in Equations (A2) and (A3) into Equation (A1), we have

(A4) $$\begin{array}{c} ∥{\hat{U}}_{c}\hat{O}-U_{c}{∥}_{F}\leq C\frac{\sqrt{K_{r}\max\left(n_{r},n_{c}\right)\log\left(n_{r}+n_{c}\right)}}{\sigma_{K_{r}}\left(\tilde{P}\right)\sqrt{\rho }\sigma_{K_{r}}\left(\Pi_{r}\right)\sqrt{n_{c,K_{r}}}}. \end{array}$$

Let ς>0 be a small quantity; by Lemma 2 in [15], if

(A5) $$\begin{array}{c} \frac{\sqrt{K_{c}}}{\varsigma }∥U_{c}-{\hat{U}}_{c}\hat{O}{∥}_{F}\left(\frac{1}{\sqrt{n_{c,k}}}+\frac{1}{\sqrt{n_{c,l}}}\right)\leq {∥B_{c}\left(k,:\right)-B_{c}\left(l,:\right)∥}_{F},\;\mathrm{for}\;\mathrm{each}\;1\leq k\neq l\leq K_{c}, \end{array}$$

then the clustering error ${\hat{f}}_{c}=O\left(\varsigma^{2}\right)$. Recall that we set $\delta_{c}=\min_{k\neq l}{∥B_{c}\left(k,:\right)-B_{c}\left(l,:\right)∥}_{F}$ to measure the minimum center separation of $B_{c}$. Setting $\varsigma =\frac{2}{\delta_{c}}\sqrt{\frac{K_{c}}{n_{c,\min}}}{∥U_{c}-{\hat{U}}_{c}\hat{O}∥}_{F}$ makes Equation (A5) hold for all $1\leq k\neq l\leq K_{c}$. Then, we have ${\hat{f}}_{c}=O\left(\varsigma^{2}\right)=O\left(\frac{K_{c}{∥U_{c}-{\hat{U}}_{c}\hat{O}∥}_{F}^{2}}{\delta_{c}^{2}n_{c,\min}}\right)$. By Equation (A4), we have

$$\begin{array}{c} {\hat{f}}_{c}=O\left(\frac{K_{r}K_{c}\max\left(n_{r},n_{c}\right)\log\left(n_{r}+n_{c}\right)}{\sigma_{K_{r}}^{2}\left(\tilde{P}\right)\rho \delta_{c}^{2}\sigma_{K_{r}}^{2}\left(\Pi_{r}\right)n_{c,K_{r}}n_{c,\min}}\right). \end{array}$$

Especially, when $K_{r}=K_{c}=K$, $\delta_{c}\geq \sqrt{\frac{2}{n_{c,\max}}}$ under $ONM_{n_{r},n_{c}}\left(K_{r},K_{c},P,\Pi_{r},\Pi_{c}\right)$ by Lemma 1. When $K_{r}=K_{c}=K$, we have

$$\begin{array}{c} {\hat{f}}_{c}=O\left(\frac{K^{2}\max\left(n_{r},n_{c}\right)n_{c,\max}\log\left(n_{r}+n_{c}\right)}{\sigma_{K}^{2}\left(\tilde{P}\right)\rho \sigma_{K}^{2}\left(\Pi_{r}\right)n_{c,\min}^{2}}\right). \end{array}$$

□

### Appendix B.4. Proof of Corollary 1

**Proof.** For the row nodes, under the conditions of Corollary 1, we have

$$\begin{array}{c} \max_{i_{r}\in \left[n_{r}\right]}{∥e_{i_{r}}^{\prime }\left({\hat{\Pi }}_{r}-\Pi_{r}P_{r}\right)∥}_{1}=O\left(\varpi K_{r}\sqrt{\frac{n_{r}}{K_{r}}}\right)=O\left(\varpi \sqrt{Kn_{r}}\right). \end{array}$$

Under the conditions of Corollary 1, κ(Ω)=O(1) and μ≤C for some C>0 by the proof of Corollary 1 [21]. Then, by Lemma A1, we have

$$\begin{array}{cc} \varpi & =O\left(\frac{\sqrt{K}\left(\kappa \left(\Omega \right)\sqrt{\frac{\max(n_{r},n_{c})\mu }{\min(n_{r},n_{c})}}+\sqrt{\log(n_{r}+n_{c})}\right)}{\sqrt{\rho }\sigma_{K}\left(\tilde{P}\right)\sigma_{K}\left(\Pi_{r}\right)\sqrt{n_{c,K_{r}}}}\right)=O\left(\frac{\sqrt{K}\left(\sqrt{\frac{C\max(n_{r},n_{c})}{\min(n_{r},n_{c})}}+\sqrt{\log(n_{r}+n_{c})}\right)}{\sqrt{\rho }\sigma_{K}\left(\tilde{P}\right)\sigma_{K}\left(\Pi_{r}\right)\sqrt{n_{c,\min}}}\right)\\ \; & =O(\frac{K^{1.5}\left(\sqrt{\frac{C\max(n_{r},n_{c})}{\min(n_{r},n_{c})}}+\sqrt{\log(n_{r}+n_{c})}\right)}{\sigma_{K}\left(\tilde{P}\right)\sqrt{\rho n_{r}n_{c}}}), \end{array}$$

which gives that

$$\begin{array}{c} \max_{i_{r}\in \left[n_{r}\right]}{∥e_{i_{r}}^{\prime }\left({\hat{\Pi }}_{r}-\Pi_{r}P_{r}\right)∥}_{1}=O\left(\frac{K^{2}\left(\sqrt{\frac{C\max(n_{r},n_{c})}{\min(n_{r},n_{c})}}+\sqrt{\log(n_{r}+n_{c})}\right)}{\sigma_{K}\left(\tilde{P}\right)\sqrt{\rho n_{c}}}\right). \end{array}$$

Note that, when $K_{r}<K_{c}$, we cannot draw a conclusion that μ≤C. This is because, when $K_{r}<K_{c}$, the inverse of $B_{c}B_{c}^{\prime }$ does not exist, since $B_{c}\in R^{K_{c}\times K_{r}}$. Therefore, Lemma 8 of [21] does not hold, and we cannot obtain the upper bound of $∥U_{c}{∥}_{2\to \infty }$, causing the impossibility of obtaining the upper bound of μ, and this is the reason for why we only consider the case for when $K_{r}=K_{c}$, for the row nodes here. For the column nodes, under the conditions of Corollary 1, we have

$$\begin{array}{cc} {\hat{f}}_{c} & =O\left(\frac{K_{r}K_{c}\max\left(n_{r},n_{c}\right)\log\left(n_{r}+n_{c}\right)}{\sigma_{K_{r}}^{2}\left(\tilde{P}\right)\rho \delta_{c}^{2}\sigma_{K_{r}}^{2}\left(\Pi_{r}\right)n_{c,K_{r}}n_{c,\min}}\right)=O\left(\frac{K_{r}K_{c}\max\left(n_{r},n_{c}\right)\log\left(n_{r}+n_{c}\right)}{\sigma_{K_{r}}^{2}\left(\tilde{P}\right)\rho \delta_{c}^{2}\left(n_{r}/K_{r}\right)\left(n_{c}/K_{c}\right)\left(n_{c}/K_{c}\right)}\right)\\ \; & =O(\frac{K_{r}^{2}K_{c}^{3}\max\left(n_{r},n_{c}\right)\log\left(n_{r}+n_{c}\right)}{\sigma_{K_{r}}^{2}\left(\tilde{P}\right)\rho \delta_{c}^{2}n_{r}n_{c}^{2}}). \end{array}$$

For the special case $K_{r}=K_{c}=K$, since $\frac{n_{c,\max}}{n_{c,\min}}=O\left(1\right)$ when $n_{c,\min}=O\left(\frac{n_{c}}{K}\right)$, we have

$$\begin{array}{c} {\hat{f}}_{c}=O\left(\frac{K^{4}\max\left(n_{r},n_{c}\right)\log\left(n_{r}+n_{c}\right)}{\sigma_{K}^{2}\left(\tilde{P}\right)\rho n_{r}n_{c}}\right). \end{array}$$

When $n_{r}=O\left(n\right),n_{c}=O\left(n\right),K_{r}=O\left(1\right)$, and $K_{c}=O\left(1\right)$, the corollary follows immediately by basic algebra. □

## Appendix C. Proofs under ODCNM

### Appendix C.1. Proof of Proposition 2

**Proof.** Since $\Omega =\Pi_{r}P\Pi_{c}^{\prime }\Theta_{c}={\overset{ˇ}{\Pi }}_{r}\overset{ˇ}{P}{\overset{ˇ}{\Pi }}_{c}^{\prime }{\overset{ˇ}{\Theta }}_{c}=U_{r}\Lambda U_{c}^{\prime }$, we have $U_{r}=\Pi_{r}U_{r}\left(I_{r},:\right)={\overset{ˇ}{\Pi }}_{r}U_{r}\left(I_{r},:\right)$ by Lemma 2, which gives that $\Pi_{r}={\overset{ˇ}{\Pi }}_{r}$. Since $U_{c,*}=\Pi_{c}B_{c}=\Pi_{c}U_{c,*}\left(I_{c},:\right)={\overset{ˇ}{\Pi }}_{c}U_{c,*}\left(I_{c},:\right)$ by Lemma 2, we have $\Pi_{c}={\overset{ˇ}{\Pi }}_{c}$. □

### Appendix C.2. Proof of Lemma 2

**Proof.**  

- For $U_{r}$: since $\Omega =U_{r}\Lambda U_{c}^{\prime }$ and $U_{c}^{\prime }U_{c}=I_{K_{r}}$, we have $U_{r}=\Omega U_{c}\Lambda^{-1}$. Recall that $\Omega =\Pi_{r}P\Pi_{c}^{\prime }\Theta_{c}$ under ODCNM; we have $U_{r}=\Pi_{r}P\Pi_{c}^{\prime }\Theta_{c}U_{c}\Lambda^{-1}=\Pi_{r}B_{r}$, where $B_{r}=P\Pi_{c}^{\prime }\Theta_{c}U_{c}\Lambda^{-1}$. Sure, $U_{r}\left(i_{r},:\right)=U_{r}\left({\bar{i}}_{r},:\right)$ holds when $\Pi_{r}\left(i_{r},:\right)=\Pi_{r}\left({\bar{i}}_{r},:\right)$ for $i_{r},{\bar{i}}_{r}\in \left[n_{r}\right]$.
- For $U_{c}$: let $D_{c}$ be a $K_{c}\times K_{c}$ diagonal matrix, such that $D_{c}\left(k,k\right)=\frac{∥\Theta_{c}\Pi_{c}{\left(:,k\right)∥}_{F}}{∥\theta_{c}{∥}_{F}}$ for $k\in [K_{c}]$. Let $\Gamma_{c}$ be an $n_{c}\times K_{c}$ matrix, such that $\Gamma_{c}\left(:,k\right)=\frac{\Theta_{c}\Pi_{c}\left(:,k\right)}{∥\Theta_{c}\Pi_{c}{\left(:,k\right)∥}_{F}}$ for $k\in [K_{c}]$. For such $D_{c}$ and $\Gamma_{c}$, we have $\Gamma_{c}^{\prime }\Gamma_{c}=I_{K_{c}}$ and $\Omega =\Pi_{r}P{∥\theta_{c}∥}_{F}D_{c}\Gamma_{c}^{\prime }$, i.e., $\Theta_{c}\Pi_{c}={∥\theta_{c}∥}_{F}\Gamma_{c}D_{c}$.
  Since $\Omega =U_{r}\Lambda U_{c}^{\prime }$ and $U_{r}^{\prime }U_{r}=I_{K_{r}}$, we have $U_{c}=\Theta_{c}\Pi_{c}P^{\prime }\Pi_{r}^{\prime }U_{r}\Lambda^{-1}$. Since $\Theta_{c}\Pi_{c}={∥\theta_{c}∥}_{F}\Gamma_{c}D_{c}$, we have $U_{c}=\Gamma_{c}{∥\theta_{c}∥}_{F}D_{c}P^{\prime }\Pi_{r}^{\prime }U_{r}\Lambda^{-1}=\Gamma_{c}V_{c}$, where we set
  $V_{c}={∥\theta_{c}∥}_{F}D_{c}P^{\prime }\Pi_{r}^{\prime }U_{r}\Lambda^{-1}\in R^{K_{c}\times K_{r}}$. Note that since $U_{c}^{\prime }U_{c}=I_{K_{r}}=V_{c}^{\prime }\Gamma_{c}^{\prime }\Gamma_{c}V_{c}=V_{c}^{\prime }V_{c}$, we have $V_{c}^{\prime }V_{c}=I_{K_{r}}$. Now, for $i_{c}\in \left[n_{c}\right],k\in \left[K_{r}\right]$, we have
  $$\begin{array}{cc} U_{c}\left(i_{c},k\right) & =e_{i_{c}}^{\prime }U_{c}e_{k}=e_{i_{c}}^{\prime }\Gamma_{c}V_{c}e_{k}=\Gamma_{c}\left(i_{c},:\right)V_{c}e_{k}\\ \; & =\theta_{c}\left(i_{c}\right)\left[\frac{\Pi_{c}\left(i_{c},1\right)}{∥\Theta_{c}\Pi_{c}{\left(:,1\right)∥}_{F}}\;\;\frac{\Pi_{c}\left(i_{c},2\right)}{∥\Theta_{c}\Pi_{c}{\left(:,2\right)∥}_{F}}\;\;\ldots \;\;\frac{\Pi_{c}\left(i_{c},K_{c}\right)}{∥\Theta_{c}\Pi_{c}\left(:,K_{c}\right){∥}_{F}}\right]V_{c}e_{k}\\ \; & =\frac{\theta_{c}\left(i_{c}\right)}{∥\Theta_{c}\Pi_{c}\left(:,\ell \left(i_{c}\right)\right){∥}_{F}}V_{c}\left(\ell \left(i_{c}\right),k\right), \end{array}$$
  which gives that
  $$\begin{array}{cc} U_{c}\left(i_{c},:\right) & =\frac{\theta_{c}\left(i_{c}\right)}{∥\Theta_{c}\Pi_{c}{(:,\ell \left(i_{c}\right)∥}_{F}}\left[V_{c}\left(\ell \left(i_{c}\right),1\right)\;\;V_{c}\left(\ell \left(i_{c}\right),2\right)\;\;\ldots \;\;V_{c}\left(\ell \left(i_{c}\right),K_{r}\right)\right]\\ \; & =\frac{\theta_{c}\left(i_{c}\right)}{∥\Theta_{c}\Pi_{c}{(:,\ell \left(i_{c}\right)∥}_{F}}V_{c}\left(\ell \left(i_{c}\right),:\right). \end{array}$$
  Then, we have
  (A6) $$\begin{array}{c} U_{c,*}\left(i_{c},:\right)=\frac{V_{c}\left(\ell \left(i_{c}\right),:\right)}{∥V_{c}\left(\ell \left(i_{c}\right),:\right){∥}_{F}}. \end{array}$$
  Sure, we have $U_{c,*}\left(i_{c},:\right)=U_{c,*}\left({\bar{i}}_{c},:\right)$ when $\ell \left(i_{c}\right)=\ell \left({\bar{i}}_{c}\right)$ for $i_{c},{\bar{i}}_{c}\in \left[n_{c}\right]$. Let $B_{c}\in R^{K_{c}\times K_{r}}$, such that $B_{c}\left(l,:\right)=\frac{V_{c}\left(l,:\right)}{∥V_{c}{\left(l,:\right)∥}_{F}}$ for $l\in [K_{c}]$. Equation (A6) gives $U_{c,*}=\Pi_{c}B_{c}$, which guarantees the existence of $B_{c}$.
  Now, we consider the case for when $K_{r}=K_{c}=K$. Since $V_{c}\in R^{K_{c}\times K_{r}}$ and $U_{c}=\Gamma_{c}V_{c}\in R^{n_{c}\times K_{r}}$, we have $V_{c}\in R^{K\times K}$ and $\mathrm{rank}(V_{c})=K$. Since $V_{c}^{\prime }V_{c}=I_{K_{r}}$, we have $V_{c}^{\prime }V_{c}=I_{K}$ when $K_{r}=K_{c}=K$. Then, we have
  (A7) $$\begin{array}{c} V_{c}^{\prime }V_{c}=I_{K}\Rightarrow V_{c}^{\prime }V_{c}V_{c}^{\prime }=V_{c}^{\prime }\Rightarrow V_{c}^{\prime }\left(V_{c}V_{c}^{\prime }-I_{K}\right)=0\overset{\mathrm{rank}(V_{c})=K}{\Rightarrow }V_{c}V_{c}^{\prime }=I_{K}. \end{array}$$
  Since $V_{c}V_{c}^{\prime }=V_{c}^{\prime }V_{c}=I_{K}$, we have $U_{c,*}\left(i_{c},:\right)=V_{c}\left(\ell \left(i_{c}\right),:\right)$ by Equation (A6), and
  $∥U_{c,*}\left(i_{c},:\right)-U_{c,*}\left({\bar{i}}_{c},:\right){∥}_{F}={∥V_{c}\left(\ell \left(i_{c}\right),:\right)-V_{c}\left(\ell \left({\bar{i}}_{c}\right),:\right)∥}_{F}=\sqrt{2}$ when $\ell \left(i_{c}\right)\neq \ell \left({\bar{i}}_{c}\right)$ for $i_{c},{\bar{i}}_{c}\in \left[n_{c}\right]$, i.e., $∥B_{c}\left(k,:\right)-B_{c}{\left(l,:\right)∥}_{F}=\sqrt{2}$ for k≠l∈[K].
  Note that, when $K_{r}<K_{c}$, since $\mathrm{rank}\left(V_{c}\right)=K_{r}$ and $V_{c}\in R^{K_{c}\times K_{r}}$, the inverse of $V_{c}$ does not exist, which causes that the last equality in Equation (A7) does not hold and $∥B_{c}\left(k,:\right)-B_{c}\left(\ell ,:\right)∥\neq \sqrt{2}$ for all $k\neq l\in [K_{c}]$.

□

### Appendix C.3. Proof of Theorem 2

**Proof.** First, by the proof of Lemma 4.3 of [25], we have the below lemma.

**Lemma** **A2.**
*(Row-wise singular eigenvector error) Under $ODCNM_{n_{r},n_{c}}\left(K_{r},K_{c},P,\Pi_{r},\Pi_{c},and\Theta_{c}\right)$, when Assumption 2 holds, suppose that $\sigma_{K_{r}}\left(\Omega \right)\geq C\sqrt{\theta_{c,\max}\left(n_{r}+n_{c}\right)\log\left(n_{r}+n_{c}\right)}$, with a probability at least $1-o({\left(n_{r}+n_{c}\right)}^{-\alpha })$,*

$$\begin{array}{cc} \; & ∥{\hat{U}}_{r}{\hat{U}}_{r}^{\prime }-U_{r}U_{r}^{\prime }{∥}_{2\to \infty }=O\left(\frac{\sqrt{\theta_{c,\max}K_{r}}\left(\kappa \left(\Omega \right)\sqrt{\frac{\max(n_{r},n_{c})\mu }{\min(n_{r},n_{c})}}+\sqrt{\log(n_{r}+n_{c})}\right)}{\theta_{c,\min}\sigma_{K_{r}}\left(P\right)\sigma_{K_{r}}\left(\Pi_{r}\right)\sqrt{n_{c,K_{r}}}}\right). \end{array}$$

For the row nodes, when the conditions in Lemma A2 hold, by Theorem 2 of [21], we have

$$\begin{array}{c} \max_{i_{r}\in \left[n_{r}\right]}{∥e_{i_{r}}^{\prime }\left({\hat{\Pi }}_{r}-\Pi_{r}P_{r}\right)∥}_{1}=O\left(\varpi \kappa \left(\Pi_{r}^{\prime }\Pi_{r}\right)K_{r}\sqrt{\lambda_{1}\left(\Pi_{r}^{\prime }\Pi_{r}\right)}\right). \end{array}$$

Next, we focus on the column nodes. By the proof of Lemma 3 in [19], there is an orthogonal matrix $\hat{O}$, such that

(A8) $$\begin{array}{c} ∥{\hat{U}}_{c}\hat{O}-U_{c}{∥}_{F}\leq \frac{2\sqrt{2K_{r}}∥A-\Omega ∥}{\sqrt{\lambda_{K_{r}}\left(\Omega^{\prime }\Omega \right)}}. \end{array}$$

Under $ODCNM_{n_{r},n_{c}}\left(K_{r},K_{c},P,\Pi_{r},\Pi_{c},and\;\Theta_{c}\right)$, by Lemma 4 of [25], we have

(A9) $$\begin{array}{c} \sqrt{\lambda_{K_{r}}\left(\Omega^{\prime }\Omega \right)}\geq \theta_{c,\min}\sigma_{K_{r}}\left(P\right)\sigma_{K_{r}}\left(\Pi_{r}\right)\sqrt{n_{c,K_{r}}}. \end{array}$$

By Lemma 4.2 of [25], when Assumption 2 holds, with a probability at least $1-o({\left(n_{r}+n_{c}\right)}^{-\alpha })$, we have

(A10) $$\begin{array}{c} ∥A-\Omega ∥=O(\sqrt{\max(\theta_{c,\max}n_{r},∥\theta_{c}{∥}_{1})\log\left(n_{r}+n_{c}\right)}). \end{array}$$

Substituting the two bounds in Equations (A9) and (A10) into Equation (A8), we have

(A11) $$\begin{array}{c} ∥{\hat{U}}_{c}\hat{O}-U_{c}{∥}_{F}\leq C\frac{\sqrt{K_{r}\max(\theta_{c,\max}n_{r},∥\theta_{c}{∥}_{1})\log\left(n_{r}+n_{c}\right)}}{\sigma_{K_{r}}\left(P\right)\theta_{c,\min}\sigma_{K_{r}}\left(\Pi_{r}\right)\sqrt{n_{c,K_{r}}}}. \end{array}$$

For $i_{c}\in \left[n_{c}\right]$, by basic algebra, we have

$$\begin{array}{c} ∥{\hat{U}}_{c,*}\left(i_{c},:\right)\hat{O}-U_{c,*}\left(i_{c},:\right){∥}_{F}\leq \frac{2∥{\hat{U}}_{c}\left(i_{c},:\right)\hat{O}-U_{c}\left(i_{c},:\right){∥}_{F}}{∥U_{c}\left(i_{c},:\right){∥}_{F}}. \end{array}$$

Setting $m_{c}=\min_{1\leq i_{c}\leq n_{c}}{∥U_{c}\left(i_{c},:\right)∥}_{F}$, we have

$$\begin{array}{c} ∥{\hat{U}}_{c,*}\hat{O}-U_{c,*}{∥}_{F}=\sqrt{\sum_{i_{c}=1}^{n_{c}}{∥{\hat{U}}_{c,*}\left(i_{c},:\right)\hat{O}-U_{c,*}\left(i_{c},:\right)∥}_{F}^{2}}\leq \frac{2∥{\hat{U}}_{c}\hat{O}-U_{c}{∥}_{F}}{m_{c}}. \end{array}$$

Next, we provide the lower bounds of $m_{c}$. By the proof of Lemma 2, we have

$$\begin{array}{cc} ∥U_{c}\left(i_{c},:\right){∥}_{F} & =∥\frac{\theta_{c}\left(i_{c}\right)}{∥\Theta_{c}\Pi_{c}\left(:,\ell \left(i_{c}\right)\right){∥}_{F}}V_{c}\left(\ell \left(i_{c}\right),:\right){∥}_{F}=\frac{\theta_{c}\left(i_{c}\right)}{∥\Theta_{c}\Pi_{c}\left(:,\ell \left(i_{c}\right)\right){∥}_{F}}{∥V_{c}\left(\ell \left(i_{c}\right),:\right)∥}_{F}\\ \; & \geq \frac{\theta_{c}\left(i_{c}\right)}{∥\Theta_{c}\Pi_{c}\left(:,\ell \left(i_{c}\right)\right){∥}_{F}}m_{V_{c}}\geq \frac{\theta_{c,\min}}{\theta_{c,\max}\sqrt{n_{c,\max}}}m_{V_{c}}, \end{array}$$

where we set $m_{V_{c}}=\min_{k\in [K_{c}]}{∥V_{c}\left(k,:\right)∥}_{F}$. Note that when $K_{r}=K_{c}=K$, by the Proof of Lemma 2, we know that $V_{c}V_{c}^{\prime }=I_{K}$, which gives that $∥V_{c}{\left(k,:\right)∥}_{F}=1$ for k∈[K]; i.e., $m_{V_{c}}=1$ when $K_{r}=K_{c}=K$. However, when $K_{r}<K_{c}$, it is challenge to obtain a positive lower bound of $m_{V_{c}}$. Hence, we have $\frac{1}{m_{c}}\leq \frac{\theta_{c,\max}\sqrt{n_{c,\max}}}{\theta_{c,\min}m_{V_{c}}}$. Then, by Equation (A11), we have

$$\begin{array}{c} ∥{\hat{U}}_{c,*}\hat{O}-U_{c,*}{∥}_{F}=O\left(\frac{\theta_{c,\max}\sqrt{K_{r}\max(\theta_{c,\max}n_{r},∥\theta_{c}{∥}_{1})n_{c,\max}\log\left(n_{r}+n_{c}\right)}}{\sigma_{K_{r}}\left(P\right)\theta_{c,\min}^{2}m_{V_{c}}\sigma_{K_{r}}\left(\Pi_{r}\right)\sqrt{n_{c,K_{r}}}}\right). \end{array}$$

Let ς>0 be a small quantity; by Lemma 2 in [15], if

(A12) $$\begin{array}{c} \frac{\sqrt{K_{c}}}{\varsigma }∥U_{c,*}-{\hat{U}}_{c,*}\hat{O}{∥}_{F}\left(\frac{1}{\sqrt{n_{c,k}}}+\frac{1}{\sqrt{n_{c,l}}}\right)\leq {∥B_{c}\left(k,:\right)-B_{c}\left(l,:\right)∥}_{F},\;\mathrm{for}\;\mathrm{each}\;1\leq k\neq l\leq K_{c}, \end{array}$$

then the clustering error ${\hat{f}}_{c}=O\left(\varsigma^{2}\right)$. Setting $\varsigma =\frac{2}{\delta_{c}}\sqrt{\frac{K_{c}}{n_{c,\min}}}{∥U_{c,*}-{\hat{U}}_{c,*}\hat{O}∥}_{F}$ makes Equation (A12) hold for all $1\leq k\neq l\leq K_{c}$. Then, we have ${\hat{f}}_{c}=O\left(\varsigma^{2}\right)=O\left(\frac{K_{c}{∥U_{c,*}-{\hat{U}}_{c,*}\hat{O}∥}_{F}^{2}}{\delta_{c}^{2}n_{c,\min}}\right)$. By Equation (A11), we have

$$\begin{array}{c} {\hat{f}}_{c}=O\left(\frac{\theta_{c,\max}^{2}K_{r}K_{c}\max(\theta_{c,\max}n_{r},∥\theta_{c}{∥}_{1})n_{c,\max}\log\left(n_{r}+n_{c}\right)}{\sigma_{K_{r}}^{2}\left(P\right)\theta_{c,\min}^{4}\delta_{c}^{2}m_{V_{c}}^{2}\sigma_{K_{r}}^{2}\left(\Pi_{r}\right)n_{c,K_{r}}n_{c,\min}}\right). \end{array}$$

Especially, when $K_{r}=K_{c}=K$, $\delta_{c}=\sqrt{2}$ under $ODCNM_{n_{r},n_{c}}\left(K_{r},K_{c},P,\Pi_{r},\Pi_{c},\Theta_{c}\right)$ by Lemma 2, and $m_{V_{c}}=1$. When $K_{r}=K_{c}=K$, we have

$$\begin{array}{c} {\hat{f}}_{c}=O\left(\frac{\theta_{c,\max}^{2}K^{2}\max(\theta_{c,\max}n_{r},∥\theta_{c}{∥}_{1})n_{c,\max}\log\left(n_{r}+n_{c}\right)}{\sigma_{K}^{2}\left(P\right)\theta_{c,\min}^{4}\sigma_{K}^{2}\left(\Pi_{r}\right)n_{c,\min}^{2}}\right). \end{array}$$

□

### Appendix C.4. Proof of Corollary 2

**Proof.** For the row nodes, under the conditions of Corollary 2, we have

$$\begin{array}{c} \max_{i_{r}\in \left[n_{r}\right]}{∥e_{i_{r}}^{\prime }\left({\hat{\Pi }}_{r}-\Pi_{r}P_{r}\right)∥}_{1}=O\left(\varpi K_{r}\sqrt{\frac{n_{r}}{K_{r}}}\right)=O\left(\varpi \sqrt{Kn_{r}}\right). \end{array}$$

Under the conditions of Corollary 2, κ(Ω)=O(1) and $\mu \leq C\frac{\theta_{c,\max}^{2}}{\theta_{c,\min}^{2}}\leq C$ for some C>0 by Lemma 2 of [25]. Then, by Lemma A2, we have

$$\begin{array}{cc} \varpi & =O(\frac{\sqrt{\theta_{c,\max}K_{r}}\left(\kappa \left(\Omega \right)\sqrt{\frac{\max(n_{r},n_{c})\mu }{\min(n_{r},n_{c})}}+\sqrt{\log(n_{r}+n_{c})}\right)}{\theta_{c,\min}\sigma_{K_{r}}\left(P\right)\sigma_{K_{r}}\left(\Pi_{r}\right)\sqrt{n_{c,K_{r}}}})\\ \; & =O(\frac{\sqrt{\theta_{c,\max}K}\left(\kappa \left(\Omega \right)\sqrt{\frac{\max(n_{r},n_{c})\mu }{\min(n_{r},n_{c})}}+\sqrt{\log(n_{r}+n_{c})}\right)}{\theta_{c,\min}\sigma_{K}\left(P\right)\sigma_{K}\left(\Pi_{r}\right)\sqrt{n_{c,\min}}})\\ \; & =O(\frac{K^{1.5}\sqrt{\theta_{c,\max}}\left(\sqrt{\frac{C\max(n_{r},n_{c})}{\min(n_{r},n_{c})}}+\sqrt{\log(n_{r}+n_{c})}\right)}{\theta_{c,\min}\sigma_{K}\left(P\right)\sqrt{n_{r}n_{c}}}), \end{array}$$

which gives that

$$\begin{array}{c} \max_{i_{r}\in \left[n_{r}\right]}{∥e_{i_{r}}^{\prime }\left({\hat{\Pi }}_{r}-\Pi_{r}P_{r}\right)∥}_{1}=O\left(\frac{K^{2}\sqrt{\theta_{c,\max}}\left(\sqrt{\frac{C\max(n_{r},n_{c})}{\min(n_{r},n_{c})}}+\sqrt{\log(n_{r}+n_{c})}\right)}{\theta_{c,\min}\sigma_{K}\left(P\right)\sqrt{n_{c}}}\right). \end{array}$$

The reason for why we do not consider the case when $K_{r}<K_{c}$ for row nodes is similar as that of Corollary 1, and we omit it here. For column nodes, under conditions of Corollary 2, we have

$$\begin{array}{cc} {\hat{f}}_{c} & =O(\frac{\theta_{c,\max}^{2}K_{r}K_{c}\max(\theta_{c,\max}n_{r},∥\theta_{c}{∥}_{1})n_{c,\max}\log\left(n_{r}+n_{c}\right)}{\sigma_{K_{r}}^{2}\left(P\right)\theta_{c,\min}^{4}\delta_{c}^{2}m_{V_{c}}^{2}\sigma_{K_{r}}^{2}\left(\Pi_{r}\right)n_{c,K_{r}}n_{c,\min}})\\ \; & =O(\frac{\theta_{c,\max}^{2}K_{r}^{2}K_{c}^{2}\max(\theta_{c,\max}n_{r},∥\theta_{c}{∥}_{1})\log\left(n_{r}+n_{c}\right)}{\sigma_{K_{r}}^{2}\left(P\right)\theta_{c,\min}^{4}\delta_{c}^{2}m_{V_{c}}^{2}n_{r}n_{c}}). \end{array}$$

For the case $K_{r}=K_{c}=K$, we have

$$\begin{array}{cc} {\hat{f}}_{c} & =O(\frac{\theta_{c,\max}^{2}K^{2}\max(\theta_{c,\max}n_{r},∥\theta_{c}{∥}_{1})n_{c,\max}\log\left(n_{r}+n_{c}\right)}{\sigma_{K}^{2}\left(P\right)\theta_{c,\min}^{4}\sigma_{K}^{2}\left(\Pi_{r}\right)n_{c,\min}^{2}})\\ \; & =O(\frac{\theta_{c,\max}^{2}K^{4}\max(\theta_{c,\max}n_{r},∥\theta_{c}{∥}_{1})\log\left(n_{r}+n_{c}\right)}{\sigma_{K}^{2}\left(P\right)\theta_{c,\min}^{4}n_{r}n_{c}}). \end{array}$$

When $n_{r}=O\left(n\right),n_{c}=O\left(n\right),K_{r}=O\left(1\right)$ and $K_{c}=O\left(1\right)$, the corollary follows immediately by basic algebra. □
